# Glycoprotein-Notebook: A Pan-Cancer Glycoproteomic Database and Toolkit for Analysis of Protein Glycosylation Changes Associated With Cancer Phenotypes

**DOI:** 10.1016/j.mcpro.2025.101089

**Published:** 2025-10-13

**Authors:** Hui Zhang, Trung Hoang, Yingwei Hu

**Affiliations:** Department of Pathology, Johns Hopkins University School of Medicine, Baltimore, Maryland, USA

**Keywords:** glycoproteomics, glycopeptides, cancer, pancreatic ductal adenocarcinoma, mass spectrometry, database

## Abstract

Protein glycosylation plays a pivotal role in various biological processes, and the analysis of intact glycopeptides (IGPs) has emerged as a powerful approach for characterizing alterations in protein glycosylation associated with diseases. Despite the critical insights gained from IGP analysis, dedicated databases and specialized tools for comprehensive glycoproteomics remain scarce. In response to this deficiency, we developed "Glycoprotein-Notebook," an online resource that consolidates the mass spectrometry evidence for IGPs identified from pan-cancer types studied in the Clinical Proteomic Tumor Analysis Consortium projects and provides analytical tools for in-depth glycopeptide characterization. Using pancreatic ductal adenocarcinoma as a case study, we validated and showcased the toolkit’s analytical capabilities. Our results underscore the promise of IGPs as cancer-specific diagnostic and therapeutic targets. Accordingly, Glycoprotein-Notebook emerges as a valuable resource for cancer researchers exploring the intricate relationship between protein glycosylation and cancer phenotypes.

## Highlights

• Unified pan-cancer glycoproteomic database of 432,750 intact glycopeptides.
• Toolkit for systematic analysis of large-scale glycoproteomic data.
• Identified cancer-associated glycosylation changes in pancreatic cancer.

## Graphical Abstract

**Figure:**
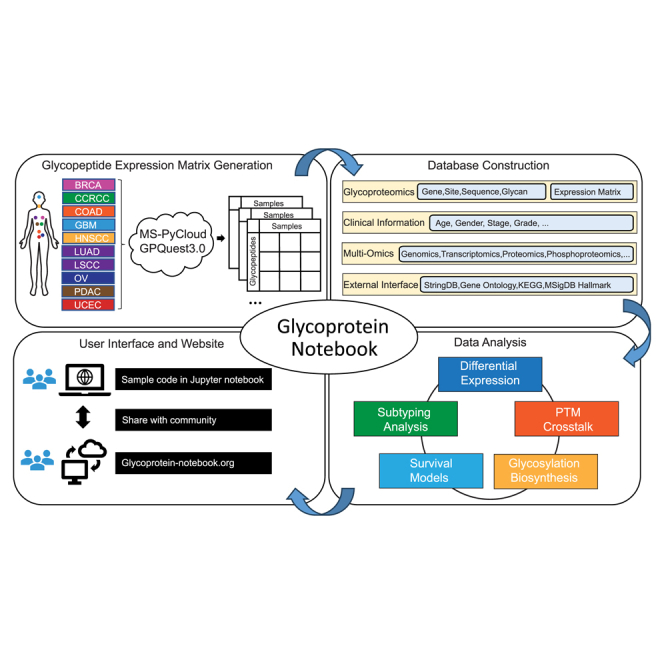

## In Brief

Glycoprotein-Notebook is an online resource integrating glycoproteomic data from multiple cancer types, offering tools for comprehensive glycoproteomic exploration. Using pancreatic ductal adenocarcinoma as a case study, it demonstrates the potential of glycoproteomic analysis to uncover cancer-specific glycoproteins.

Protein glycosylation, the attachment of carbohydrate groups to proteins, is a vital process that plays roles in various biological functions (1, 2), including protein trafficking (3), cell adhesion (4), immune response (5), and receptor binding and activation (6). However, the analysis of glycoproteins is complex because of the nontemplated nature of glycosylation, the high degree of structural diversity and isomerism among glycans, and the presence of variable occupancy of glycosites (macroheterogeneity) and multiple distinct glycan structures at each glycosite (microheterogeneity). Despite these complexities, the study of protein glycosylation is important because of its potential role in diseases and as therapeutic or diagnostic targets. Current technologies, such as mass spectrometry (MS), lectin-binding assays, and ELISAs, enable the identification and characterization of glycans and the quantification of site-specific glycosylation (7, 8, 9, 10).

Although significant progress has been made in the identification of intact glycopeptides (IGPs) using tandem MS (11, 12, 13, 14, 15) and glycan databases (16, 17, 18, 19), the high-throughput analysis of glycoproteomic data still faces considerable obstacles. One prominent challenge is the lack of specialized resources tailored for the glycoproteomic evaluation of IGPs. This deficiency hampers the seamless integration of glycoproteomic information with other “omics” datasets. Furthermore, there is a shortage of analytical tools specifically calibrated for glycoproteomic analysis that can robustly link identified glycosylation alterations to cancer phenotypes, with an emphasis on reproducibility for subsequent validation and research. Addressing these challenges is critical to unlocking the full potential of glycoproteomics in cancer and the broader understanding of disease mechanisms.

We present here a glycoproteomic analysis toolkit, Glycoprotein-Notebook, as a promising approach to high-throughput glycoproteomic data analysis for IGPs. Using the MS datasets from 10 tumor types characterized by Clinical Proteomic Tumor Analysis Consortium (CPTAC) projects, it provides unified access to identified glycopeptides of CPTAC cancer glycoproteomics data, comprising 432,750 N-linked glycopeptides from 11,641 proteins. The 10 cancer types include breast carcinoma (BRCA) (20), clear cell renal cell carcinoma (CCRCC) (21), colorectal carcinoma (COAD) (22), glioblastoma (GBM) (23), head and neck squamous cell carcinoma (HNSCC) (24), lung squamous cell carcinoma (LSCC) (25), lung adenocarcinoma (LUAD) (26), ovarian serous cystadenocarcinoma (OV) (27), pancreatic ductal adenocarcinoma (PDAC) (28), and uterine corpus endometrial carcinoma (UCEC) (29, 30). Based on standardized expression matrices, we developed Glycoprotein-Notebook for comprehensive glycoproteomic data analysis based on IGPs. The utility of the toolkit is demonstrated through its applications in statistical profiling, differential expression analysis, glycosylation enzyme assessment, glycoform-based subtyping, survival analysis, and investigation of glycosylation–phosphorylation crosstalk for a PDAC cohort.

## Experimental procedures

### Data Source

Glycoprotein-Notebook extends the CPTAC database by incorporating glycoproteomic expression matrices for primary tumors and normal adjacent samples from 10 CPTAC cancer types. Glycoprotein-Notebook provides tools for comprehensive glycoproteomic analysis and integrates the glycoproteomic data with transcriptomic, proteomic, phosphoproteomic, and clinical data for the 10 CPTAC cancer types downloaded from LinkedOmics (31) and literature sources (20, 21, 22, 23, 24, 25, 26, 27, 28, 29, 30). The clinical data included in this database cover tumor stage, gender, age, tumor site, survival information, and molecular subtyping annotation reported in the literature (Supplementary Table S1). Substage-level data were consolidated under respective main stages (I, II, III, and IV). All molecular data were properly normalized and stored in feature-by-sample matrix format, with features as rows and samples as columns.

### Database Construction and Characterization

The identification of intact N-linked glycopeptides in this study was conducted using GPQuest 3.0 software (Zhang Lab) (32, 33, 34). GPQuest 3.0 was used to identify IGPs from MS/MS spectra through three steps: (1) detecting spectra containing oxonium ions ("oxo-spectra"); (2) identifying the peptide backbone by matching *b*- and *y*-type fragment ions; and (3) assigning the IGP by matching the glycan mass and the ladder of *Y*-ion (peptide + fragments from N-linked core structure) ladder.

The oxonium ions were used as signature features of glycopeptides from MS/MS spectra, which resulted from the fragmentation of glycans attached to IGPs in the mass spectrometer. In this study, the MS/MS spectra containing oxonium ions (*m/z* 204.0966) in the top 10 most abundant peaks after removing tandem mass tag (TMT) reporter ions were considered potential glycopeptide candidates. GPQuest 3.0 was then used to identify intact N-linked glycopeptides by searching against a database of deglycosylated peptide sequences containing the N-X-S/T motif (where X ≠ proline), derived from 51,689 protein entries in GENCODE, v42 (35) and a glycan database comprising 695 N-linked glycan compositions reported in pGlyco3 (15). To estimate the false discovery rate (FDR), an equal-sized decoy set of deglycosylated peptides was generated by random shifting and searched in parallel.

Each tandem mass spectrum was first processed in a series of preprocessing procedures, including removing reporter ions, spectrum denoising, MS deisotoping (36), oxonium ion evaluation, and glycan type prediction (37). The top 300 peaks in each preprocessed spectrum were matched to the fragment ion index generated from a peptide sequence database to identify all candidate peptides. All qualified (>6 fragment ion matchings) candidate peptides were compared with the spectrum again to calculate the Morpheus scores (38) by considering all the peptide fragments, glycopeptide fragments, and their isotope peaks. The peptide with the highest Morpheus score was then assigned to the spectrum. The mass gap between the assigned peptide and the precursor mass was searched in the glycan database to find the associated glycan. Our glycan-type scoring combines three complementary signals per identification. Oxonium ion score (OXO_score) quantifies the intensity of diagnostic oxonium ions, characteristic of the candidate glycan. Isotope score (ISO_score) validates the precursor by matching the theoretical isotopic envelope of the IGP (at the observed charge state) to the experimental MS1 profile. YIons_score tallies the number and total intensity of matched core *Y* ions (peptide + HexNAc, peptide + 2HexNAc, peptide + 2HexNAc + (1–3)Hex), FlagScore fucosylation, and applies length-dependent minimum-match thresholds for quality control. Together, these orthogonal metrics—diagnostic fragments, precursor validation, and core-fragment evidence—provide the glycan structural assignment confidence while reducing glycan-level false positives. The best hits of all "oxo-spectra" were ranked by the Morpheus score in descending order. Identifications with an FDR <1% at the glycopeptide level were retained as qualified identifications. The precursor mass tolerance was set at 10 ppm, and the fragment mass tolerance was set at 20 ppm. Up to two missed cleavages are allowed for trypsin digestion. Fixed modifications included carbamidomethylation of cysteine (C) and TMT labeling at the peptide N terminus and lysine (K), whereas oxidation of methionine (M) was set as a variable modification.

Quantification of IGPs was performed at both the glycopeptide level and the glycoform level. Each glycoform was defined as a unique combination of protein ID, gene symbol, glycosylation site, and glycan composition (*e.g.*, ENSP00000009530_CD74_136_N2H8F0S0G0). A glycoform's expression equals the glycopeptide expression if only one glycopeptide covers the glycosite. If multiple glycopeptides map to the same glycosite, the glycoform expression is the sum of their intensities. Thus, a glycoform may represent one or more glycopeptides assigned to the same site. Median-normalized log_2_ ratios to the pooled samples were used for normalized quantification. Statistics on identified glycopeptides and corresponding glycoproteins are summarized in Supplementary Table S1.

### N-Linked Glycan–Type Definition

We defined five N-linked glycan types in this study to facilitate the discussion of the common trends among glycopeptides with similar monosaccharide composition, which include high mannose (HM), fucosylated only (only_F), sialylated only (only_S), fucosylated and sialylated (F + S), and all other glycans (Other). Additional details and representative glycan structures for each type are provided in Supplementary File 1.

### Gene Set Enrichment Analysis

The Python package GSEApy (gene set enrichment analysis) (39) was used to perform over-representation analysis (ORA) *via* the enrichr function. The background gene set included all genes corresponding to glycopeptides identified across all cohorts or within a specific cohort (*e.g.*, PDAC), depending on the scope of the enrichment analysis. The statistical significance threshold was typically defined as FDR <0.05, unless otherwise specified. The pathway libraries used in this study included KEGG_2021_Human, MsgDB_Hallmark_2020, GO_Biological_Process_2023, GO_Molecular_Function_2023, and GO_Cellular_Component_2023.

### Differential Expression Analysis

In order to distinguish tumors from nontumor samples, we conducted principal component analysis on the glycopeptides expressed in all tumor, normal adjacent, and normal ductal (ND) samples in the PDAC cohort using OmicsOne. In addition, we performed a Wilcoxon rank-sum test on the intact N-linked glycopeptides expressed in tumor and normal adjacent samples from the same cohort. The Benjamini–Hochberg procedure was applied to adjust *p* values, with a significance threshold set at FDR <0.01, to identify glycopeptides with altered expression as potential diagnostic markers. IGPs meeting both criteria—FDR <0.01 and an absolute log_2_ fold change (FC) ≥1 (*i.e.*, FC ≥2)—were defined as significantly upregulated (S-U) or significantly downregulated (S-D). Glycopeptides with FDR <0.01 but a log_2_ FC between −1 and 1 were categorized as upregulated (U) or downregulated (D), reflecting milder yet statistically significant changes. For the subtype-level analysis, we compared each subtype with all other tumor samples using the same differential expression methodology. The GSEApy package was employed on the S-U or S-U glycopeptides to identify the enriched pathways or Gene Ontology (GO) terms.

### Biosynthetic Glycosylation Pathway Analysis

We collected 111 glycosylation enzymes involved in the biosynthetic glycosylation pathways and investigated their RNA and protein expression levels as well as their correlations in different cancer types. The pathway diagram was downloaded from http://glycoenzymes.ccrc.uga.edu/Glycomics3/ and shown in Supplementary Table S1. The differentially expressed glycosylation enzymes between tumor and normal adjacent tissue (NAT) samples from pan-cancer cohorts were summarized and visualized according to the differential expression analysis on global proteomics data downloaded from LinkedOmics. Glycosylation in human cells is primarily mediated through three major biosynthetic pathways: the lipid-linked oligosaccharide (LLO) pathway, the N-linked glycosylation pathway (N-linked), and the common glycosylation pathway (Common), sharing terminal modifications with O-linked glycosylation and glycolipid biosynthesis. The LLO pathway initiates in the endoplasmic reticulum (ER), where a conserved oligosaccharide is assembled stepwise on a dolichol phosphate lipid carrier by a series of enzymes, including DPAGT1, DPM1–3, and ALG family members. This oligosaccharide is then transferred to polypeptides by the oligosaccharyltransferase complex, forming the precursor for N-linked glycans. The N-linked glycosylation pathway continues with glycan trimming and maturation in the ER and Golgi, where mannosidases and glycosyltransferases such as MOGS, MAN1A1, MGATs, and FUT8 generate HM, hybrid, and complex N-glycan structures. In parallel, the common glycosylation pathway assembles glycan structures through the action of specific glycosyltransferases, producing terminal modifications, such as blood group antigens, Lewis antigens, and other terminal modifications involved in cell–cell recognition and immune modulation.

To investigate the regulatory patterns of glycosylation biosynthesis in tumor samples, we performed a correlation analysis between 72 glycosylation enzymes and 192 glycan compositions across 104 tumors (sample C3N-1715.T was excluded because it is not present in the LinkedOmics protein matrix). Across all tumor samples, the median normalized log_2_ ratio values of 2000 glycoforms were Z-transformed individually and then aggregated by glycan composition using median values, resulting in a 192-glycan expression matrix for correlation analysis. Glycans were further classified into five types using HexNAc count (N) as a proxy for branching and sialylation count (S) for terminal capping: A: HM (N = 2; 5 ≤ H ≤ 12; F = 0; S = 0); B: low antenna (non-HM; N ≤ 3); C: midantenna, low sialylated (4 ≤ N ≤ 5; S ≤ 1); D: midantenna, high sialylated (4 ≤ N ≤ 5; S ≥ 2); and E: high antenna (N ≥ 6).

### Glycoform-Based Subtyping Analysis

We utilized non-negative matrix factorization (NMF)–based clustering to investigate the intratumor heterogeneity in PDAC. The procedure followed a procedure similar to our previous work on PDAC (28). Briefly, NMF was used to perform unsupervised clustering of tumor samples using the abundances of IGPs. Only features with a standard deviation greater than 25% were used for subsequent analysis. The feature matrix was scaled and standardized to *z*-scores. Since NMF requires a non-negative input matrix, the feature matrix was converted as follows: (1) created one data matrix with all negative numbers zeroed; (2) created another data matrix zeroing out all positive values and converting negative values to positive; and (3) concatenated both matrices, resulting in a data matrix with positive values and zeros only. The resulting matrix was then subjected to NMF analysis leveraging the NMF R-package (40).

To determine the optimal factorization rank *k* (*i.e.*, number of clusters), a range of *k* of 2 to 10 was evaluated using default settings with 30 iterations. The optimal factorization rank *k* = 3 was selected since the product of the cophenetic correlation coefficient and dispersion coefficient of the consensus matrix was highest at *k* = 3 compared with other tested *k* values. The NMF analysis was repeated using 500 iterations for the optimal factorization rank *k*. A list of representative features for each cluster was extracted based on the relative basis contribution to the cluster (threshold was set at 0.8).

We also evaluated the association between the clustering results derived from glycoproteome data with other subtypes, including multiomics NMF clustering results and immune cell subtypes, using the Jaccard index. To assess glycan pattern differences among subtypes, we applied cumulative distribution function analysis in combination with signed FDR statistics, enabling visualization of both statistical significance and direction of glycopeptide regulation.

We used the scikit-survival package to generate Kaplan–Meier survival curves and the lifelines package to perform statistical comparisons. Survival time and event status were used to stratify patients into IGP groups (IGP-1–3) or three groups (Low, Mid, and High) based on tertile cutoffs of the variable of interest (*e.g.*, protein or glycoform). Kaplan–Meier estimates for each group were calculated and plotted, and overall survival differences were evaluated using the multivariate_logrank_test function from lifelines.statistics, which compares survival distributions across multiple groups and returns the Chi-squared statistic and *p* value for the log-rank test.

### Glycosylation–Phosphorylation Crosstalk Analysis

We investigated the crosstalk between protein glycosylation and phosphorylation under two conditions: intraprotein and interprotein crosstalk (41). The phosphorylation datasets were collected from LinkedOmics (42).

In the intraprotein analysis, we summarized the proteins having both phosphorylation and glycosylation and visualized the overlap in a Venn diagram. Moreover, ORA was performed using GSEApy to identify significantly enriched GO terms (FDR <0.05) in proteins where both phosphorylation and glycosylation were detected, although co-occurrence on the same peptide required more evidence. Comparative ORA based on the MSigDB_Hallmark_2020 gene sets was performed for proteins with only phosphorylation, only glycosylation, and both modifications to reveal specific pathways enriched in the proteins with both glycosylation and phosphorylation under FDR <0.05. We further applied the linear regression model to demonstrate that the expression values of glycopeptides and phosphopeptides of the identical proteins were positively proportional (*p* value < 0.01).

In order to further investigate the relationship between protein glycosylation and phosphorylation across different proteins (interprotein level), the correlations between protein glycosylation and protein phosphorylation in the CPTAC PDAC 44 paired samples respectively to identify the dysregulated correlations between tumor and NAT samples. A linear regression model was employed to evaluate the overall trend. The most significantly altered correlations were selected, and gene names from the corresponding glycosites and phosphosites were extracted and submitted to GSEApy for ORA using the Kyoto Encyclopedia of Genes and Genomes (KEGG) pathway database. Enriched pathways were visualized through the KEGG web graphical user interface (https://www.kegg.jp) to illustrate protein–protein interactions within each pathway.

## Results

### Landscape of the Glycoproteomic Database

**Fig. 1:**
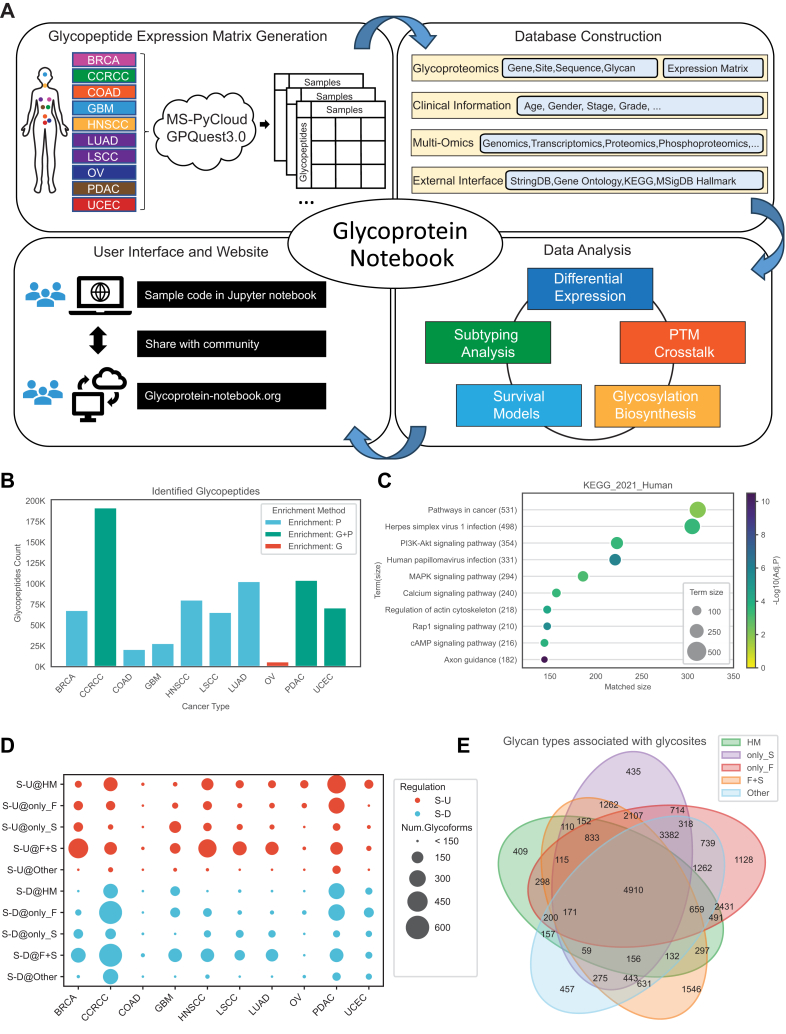
**Landscape of the glycoproteomic database.***A*, workflow of Glycoprotein-Notebook. The workflow begins with glycopeptide expression matrix generation from multiple cancer types using MS-PyCloud with GPQuest 3.0. These data are incorporated into a database containing glycoproteomics features (gene, site, sequence, and glycan), clinical information, multiomics datasets, and external annotations (*e.g.*, StringDB, Gene Ontology, KEGG, MSigDB). Precomputed analyses include differential expression, subtyping, survival modeling, PTM crosstalk, and glycosylation biosynthesis profiling. The data and analysis tools are shared with the community, and users can explore the Glycoprotein-Notebook resource at glycoprotein-notebook.org. *B*, statistics of identified glycopeptides from 10 CPTAC cohorts. Bar plot showing the total number of glycopeptides identified for each cancer type (BRCA, CCRCC, COAD, GBM, HNSCC, LSCC, LUAD, OV, PDAC, and UCEC). Bar colors indicate the enrichment method used: phosphorylation enrichment (P, *cyan*), combined glycosylation and phosphorylation enrichment (G + P, *green*), or glycosylation-specific enrichment (G, *red*). *C*, enrichment analysis of all glycoproteins in the KEGG_2021_Human pathway database. Bubble plot showing enriched pathways from the KEGG_2021_Human database. The *x*-axis represents the matched size (number of glycoproteins overlapping the gene sets), and the *y*-axis lists the significantly enriched pathways and their corresponding term size. Bubble size also indicates the term size of each pathway, and bubble color represents statistical significance as −log_10_(FDR). *D*, differential expression patterns of glycoforms across cancer types. Bubble plot showing significantly upregulated (S-U, *red*) and downregulated (S-D, *blue*) glycopeptides in 10 cancer types, stratified by five glycan types (HM, only_F, only_S, F + S, and Other). Bubble size represents the number of significantly altered glycoforms, with larger bubbles indicating higher counts. *E*, association of glycan types with glycosites across 10 cancer types. Venn diagram showing the overlap of glycosites associated with five glycan types: HM (*green*), sialylated only (only_S, *purple*), fucosylated only (only_F, *red*), both fucosylated and sialylated (F + S, *orange*), and other types (*blue*). Numbers indicate the count of glycosites within each category or intersection. BRCA, breast carcinoma; CCRCC, clear cell renal cell carcinoma; COAD, colorectal carcinoma; CPTAC, Clinical Proteomic Tumor Analysis Consortium; FDR, false discovery rate; GBM, glioblastoma; HM, high mannose; HNSCC, head and neck squamous cell carcinoma; LSCC, lung squamous cell carcinoma; LUAD, lung adenocarcinoma; OV, ovarian serous cystadenocarcinoma; PDAC, pancreatic ductal adenocarcinoma; PTM, post-translational modification; UCEC, uterine corpus endometrial carcinoma.

We collected raw MS files from 10 CPTAC cohorts, including BRCA, CCRCC, COAD, GBM, HNSCC, LUAD, LSCC, OV, PDAC, and UCEC, to construct the database for Glycoprotein-Notebook (Fig. 1*A*). Phosphoproteomic datasets are known to contain a substantial portion of glycopeptide spectra, particularly IGPs with sialic acids, which are often coenriched with phosphopeptides (32). Therefore, both glycoproteomic and phosphoproteomic datasets from tumor and NAT samples of the 10 CPTAC cancer types were used for IGP identification using MS-PyCloud (Zhang Lab, Johns Hopkins University) and GPQuest 3.0. Identified glycopeptides were aggregated at the glycoform level. We identified 432,750 N-linked IGPs (sequence + glycan), 408,728 glycoforms, 26,277 glycosites, and 11,641 glycoproteins from the 10 cancer types, all under a glycopeptide-spectrum match FDR threshold of 0.01. The corresponding clinical information—including stage, grade, and somatic mutations—was curated from the literature (20, 21, 22, 23, 24, 25, 26, 27, 28, 29, 31, 43) and integrated into the database alongside multiomics data, including genomics, transcriptomics, proteomics, and phosphoproteomics, to facilitate multidimensional analyses (*e.g.*, post-translational modification [PTM] crosstalk analysis). Users can utilize access and run the Jupyter notebooks available in the Glycoprotein-Notebook GitHub repository (https://github.com/huizhanglab-jhu/glycoproteinnotebook) and download the corresponding datasets from https://glycoprotein-notebook.org to reproduce the results or apply the workflow to their own datasets.

We identified 68,106 glycopeptides from BRCA, 191,746 from CCRCC, 21,107 from COAD, 28,285 from GBM, 80,619 from HNSCC, 102,888 from LUAD, 65,747 from LSCC, 6100 from OV, 104,412 from PDAC, and 71,197 from UCEC (Fig. 1*B* and Supplementary Table S1). The number of identified N-linked glycopeptides varied across cancer types and experimental conditions, influenced by factors such as sample fractionation, enrichment strategies, and instrument platforms. In general, increasing the number of fractions and integrating both phosphorylation- and glycosylation-enriched datasets improves identification yield. Remarkably, the ovarian cancer samples in this study include only three replicates per TMT set and lacked fractionation, resulting in a relatively lower number of identifications.

To compare glycopeptides identified through glycoenrichment and phosphoenrichment methods, we analyzed the distribution of glycan types across both datasets. Glycopeptides containing both F + S were more prevalent in the phospho-enriched data (51–55%) compared with the glyco-enriched data (22–27%). Conversely, a higher proportion of HM glycans was observed in the glyco-enriched dataset (15–27%) relative to the phospho-enriched dataset (4–6%), indicating method-dependent glycan type biases (Supplementary Fig. S1, *A* and *B*). To assess the complementarity and consistency between two enrichment methods, we compared the number of identifications at multiple levels, including genes, glycosites, peptides, and glycopeptides (Supplementary Fig. S1*C*). While each method uniquely identified a substantial number of entries, a notable overlap was observed, underscoring their complementary nature. Furthermore, Spearman's correlation analysis of overlapping glycoforms revealed a strong overall concordance between the two datasets, with a median correlation of 0.57 (Supplementary Fig. S1*D*). Based on this consistency, we integrated the glyco-enriched and phospho-enriched datasets for downstream analyses.

To identify pathways enriched among the glycoproteins, we conducted ORA using GSEApy (39) on the gene symbols of all the identified glycopeptides, referencing the human KEGG pathway database, KEGG_2021_Human (18, 44, 45). To highlight the dominant glycoproteins, we retained enriched terms with an adjusted *p* value (FDR) <0.01 and ranked them in descending order of overlapped gene-set size. KEGG pathway enrichment of the identified glycoproteins across all cancer types revealed strong enrichment in pathways fundamentally associated with tumor biology (Fig. 1*C*). Pathways in cancer showed the strongest enrichment, reflecting the central roles of glycoproteins in oncogenic processes. As an umbrella pathway, pathways in cancer integrate multiple cancer–associated signaling cascades (*e.g.*, PI3K-Akt, MAPK, Wnt, and p53), many of which were also individually enriched in our analysis. In addition, several KEGG pathways annotated as viral infections (*e.g.*, herpes simplex virus 1 infection, human papillomavirus infection, and coronavirus disease) were enriched. These do not indicate direct viral involvement but rather reflect the extensive participation of host glycoproteins in immune response, receptor-mediated entry, and signal transduction—processes that viruses often exploit. Beyond these, glycoproteins were also significantly enriched in multiple signaling pathways, including calcium signaling, Rap1, cAMP, and actin cytoskeleton regulation, highlighting their broad roles in cell communication, migration, and structural regulation. Interestingly, the axon guidance pathway exhibited the strongest enrichment among glycoproteins. This can be explained by the fact that many axon guidance molecules are glycoproteins localized at the cell surface. While originally characterized in neural development, these molecules are frequently repurposed in cancer to regulate cell adhesion, migration, and invasion. In addition, because our cohort includes GBM, tissue context likely amplifies this signal. Thus, the strong enrichment of axon guidance may reflect both the central roles of glycoproteins in mediating cell–cell and cell–matrix interactions critical for tumor progression and the neural tissue composition of the cohort. Together, these results underscore that glycoproteins are deeply embedded not only in cancer-specific signaling but also in host defense and cellular regulatory networks that are critical to tumor progression, migration, invasion, and metastasis (46, 47, 48, 49, 50).

In Figure 1*D*, we present the upregulated and downregulated glycoforms in tumors compared with nontumor samples across the 10 CPTAC cancer cohorts, categorized into five glycan structural types. The results reveal tumor-specific glycosylation patterns. CCRCC exhibits the most prominent increase in HM glycoforms, alongside a notable downregulation of fucosylated (F) and F + S species (Supplementary Table S2). LUAD, LSCC, and HNSCC display similar patterns of glycosylation alterations, suggesting shared regulatory features. PDAC also displayed strong glycoform remodeling, with both upregulation and downregulation observed in the HM and only_F categories. Furthermore, we investigated the associations between glycosites and glycans and found that approximately 85% of glycosites were modified by more than one type of N-linked glycans (Fig. 1*E*), indicating that glycan microheterogeneity is prevalent across most N-linked glycosites. This finding provides important insights for future investigations into glycosite-specific glycosylation variability.

### Differential Expression of N-Linked Glycoforms Between Tumor and NAT Samples in PDAC for Potential Diagnostic Biomarker

**Fig. 2:**
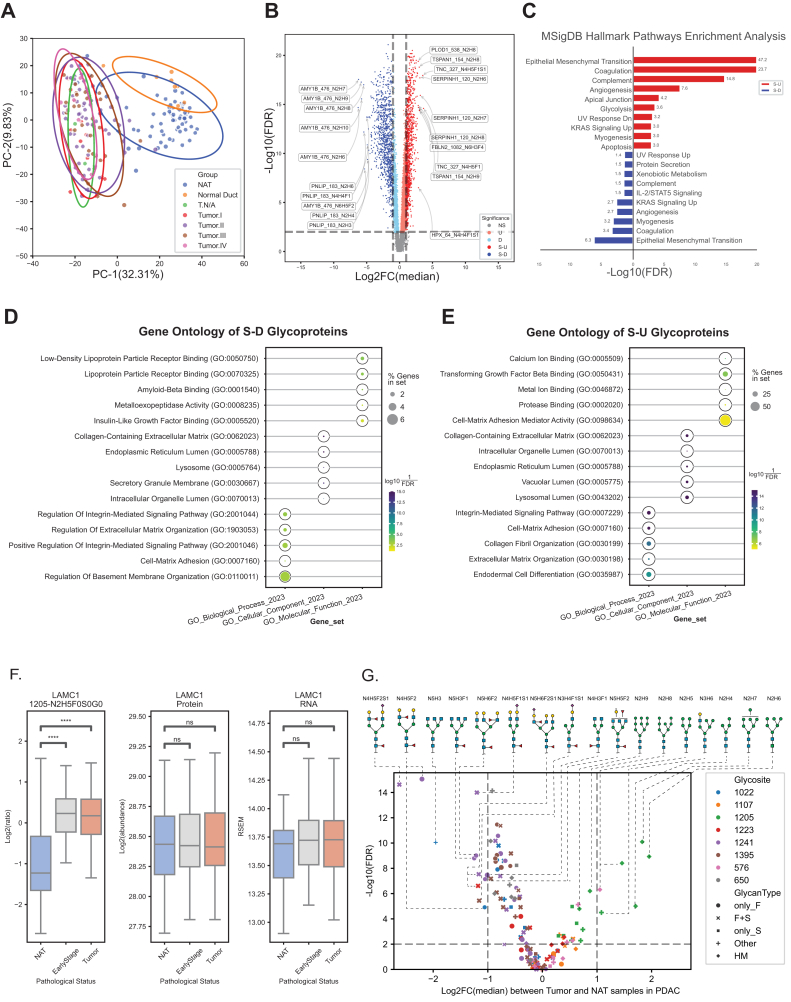
**Differential expression of N-linked glycopeptides between tumor and NAT samples in PDAC.***A*, principal component analysis of tumor, NAT, and normal duct (ND) samples with stage information. Each point represents a sample, with *ellipses* indicating 95% confidence intervals for each group. PC1 and PC2 explain 32.31% and 9.83% of the variance, respectively. *B*, volcano plot of differentially expressed glycoforms in the tumor compared with NATs. Each point represents a glycoform, with the *x*-axis showing the log_2_ fold change (median) and the *y*-axis showing −log_10_(FDR). *Light red and light blue* indicate glycoforms with FDR <0.01, whereas *dark red and dark blue* indicate glycoforms with both FDR <0.01 and |log_2_FC| >1. *Gray points* represent nonsignificant (NS) changes. Selected top glycoforms are labeled with Gene_Glycosite_GlycanComposition. *C*, hallmark pathways enriched in upregulated and downregulated glycoforms. MSigDB Hallmark pathways significantly enriched among significantly upregulated (S-U, *red*) and significantly downregulated (S-D, *blue*) glycoforms are shown. Bar length indicates enrichment significance (−log_10_FDR), and numbers at the bar ends represent the significance score. Positive values denote enrichment in S-U, and negative values denote enrichment in S-D. *D* and *E*, Gene Ontology (GO) enrichment of differentially expressed glycoforms in tumors. *D*, GO terms significantly enriched among downregulated glycoforms (S-D). *E*, GO terms significantly enriched among upregulated glycoforms (S-U). Circle size represents the percentage of genes in each term, and color denotes enrichment significance (−log_10_FDR). Reference GO gene sets are Biological Process, Cellular Component, and Molecular Function. *F*, multiomics expression profiles of TANDTSTEAYNLLLR-N2H5F0S0G0@LAMC1 in NATs, early stage (I and II) tumors, and all tumors at three molecular levels: IGP (*left*), protein (*middle*), and RNA (*right*). Statistical significance was assessed by *t*-tested pairwise comparisons. *p* Value annotation legend: ns, 0.05 < *p* ≤ 1.00; ∗0.01 < *p* ≤ 0.05; ∗∗0.001 < *p* ≤ 0.01; ∗∗∗0.0001 < *p* ≤ 0.001; ∗∗∗∗*p* ≤ 0.0001. *G*, differential expression of LAMC1 glycoforms between tumor and NAT samples. Volcano plot showing log_2_ FC *versus* –log_10_(FDR) for individual LAMC1 glycoforms, with point shapes indicating glycan types (only_F, F + S, only_S, Other, and HM) and colors representing glycosylation sites. Representative glycan structures are illustrated above, with *dashed lines* linking each structure to its corresponding data point. FC, fold change; FDR, false discovery rate; HM, high mannose; IGP, intact glycopeptide; NAT, normal adjacent tissue; PC, principal component; PDAC, pancreatic ductal adenocarcinoma.

Differentially expressed glycoforms (DEGFs) for each cancer type can be accessed in Supplementary Table S2. We integrated OmicsOne (28) to establish a pipeline for identifying DEGFs between tumor and NAT samples in various datasets, characterizing the properties of DEGFs, and exploring their association with clinical information. Pancreatic cancer remains a leading cause of cancer-related mortality globally, and early detection is crucial for improving survival rates. Our principal component analysis results, depicted in Figure 2*A*, indicate that the differential expression of glycopeptides can effectively distinguish tumor, NAT, and ND samples in most cases. Even early-stage samples (stage I and II) were distinguishable from NAT samples, suggesting the potential of glycopeptides as early detection biomarkers for PDAC.

We identified 2019 DEGFs in the comparison between 105 PDAC samples and 67 NAT samples from the PDAC dataset using the differential expression analysis *via* OmicsOne. Among them, 981 were S-D and 1038 were S-U glycoforms (Fig. 2*B*). The volcano plot illustrates significant differences in glycopeptide expression between tumor and NAT samples. Glycopeptides derived from pancreatic digestive enzymes (*e.g.*, PNLIP, AMY1B) are markedly downregulated in tumors, whereas those from extracellular matrix (ECM) and stress-related proteins (*e.g.*, TNC, SERPINH1, TSPAN1) are significantly upregulated. Glycoform-level information further reveals tumor-specific glycosylation remodeling, reinforcing the potential of glycopeptides as biomarkers for PDAC. Enriched MSigDB_Hallmark_2020 pathways among upregulated and downregulated glycopeptides are shown in Figure 2*C*. Glycoproteins involved in epithelial–mesenchymal transition (EMT), coagulation, and complement pathways were significantly enriched among the upregulated glycopeptides, underscoring their roles in tumor progression, invasion, and immune modulation. The bidirectional enrichment of several pathways suggests a complex, context-specific regulation of protein glycosylation in PDAC.

We further analyzed the GO terms enriched in the gene names corresponding to the S-D and S-U glycopeptides (Fig. 2, *D* and *E*). For S-D glycoproteins, the top enriched GO molecular-function (GO_MF) terms included low-density lipoprotein particle receptor binding, lipoprotein particle receptor binding, and amyloid-beta binding, all of which are associated with lipid metabolism and transport. Additional enriched terms, such as metalloexopeptidase activity, reflect hydrolase functions, and insulin-like growth factor binding, which point to regulation of growth factor availability and signaling. Enriched cellular-component (GO_CC) terms included the collagen-containing ECM, ER lumen, secretory-granule membrane, lysosome, and intracellular organelle lumen, whereas enriched biological-process (GO_BP) terms highlighted regulation of integrin-mediated signaling, ECM organization, cell–matrix adhesion, and regulation of basement membrane organization. Collectively, these functions reflect the secretory and metabolic activities of normal pancreatic acinar cells.

In contrast, S-U glycoproteins were enriched for GO_MF terms such as transforming growth factor-β binding, protease binding, calcium–ion binding, metal–ion binding, and cell–matrix adhesion mediator activity—functions associated with growth-factor signaling, adhesion, and protease regulation. Complementary CC terms (*e.g.*, collagen-containing ECM, ER, and intracellular-organelle lumina, vacuolar and lysosomal lumina) and BP terms (*e.g.*, ECM organization, cell–matrix adhesion, integrin-mediated signaling, and endodermal cell differentiation) indicate extensive ECM remodeling and secretory activity of PDAC cells. Taken together, the enrichment patterns observed in S-U glycoproteins—highlighting growth-factor binding, protease regulation, and extensive ECM remodeling—are consistent with the molecular hallmarks of tumor and tumor-associated stromal cells, rather than the secretory and metabolic functions typical of normal pancreatic tissue.

In our comparative analysis with the corresponding proteomic data for global protein expression, we observed that 28 of the 205 proteins linked to the 1038 S-U glycopeptides were not upregulated at the global protein level (FDR <0.01) (Supplementary Table S2). These findings suggest that glycopeptide expression may offer valuable insights for the early detection and diagnosis of PDAC, surpassing the information provided by traditional protein and mRNA expression data. Figure 2*F* presents the expression profiles of a representative glycopeptide—TANDTSTEAYNLLLR-N2H7F0S0G0@LAMC1—across NAT, tumor, and early-stage tumor samples, showing distinct upregulation at the glycoform level. The differential expression analysis of all LAMC1 glycopeptides (Fig. 2*G*) revealed substantial remodeling of glycosylation in tumors compared with NAT samples, encompassing both upregulated and downregulated glycoforms. At site N1205, HM-type glycans were markedly upregulated in tumors, whereas at site 1241, F + S complex–type glycans were downregulated, indicating distinct site-specific remodeling patterns and differential regulation of glycan processing across LAMC1 glycosites.

The regulation of protein glycosylation is governed by a complex network of enzymes that mediate the addition and removal of specific carbohydrate moieties. Glycosyltransferases catalyze the attachment of glycans to proteins, whereas glycosidases hydrolyze glycosidic bonds to remove carbohydrate residues. In this study, we investigated the expression of 111 enzymes involved in protein glycosylation biosynthesis (http://glycoenzymes.ccrc.uga.edu/Glycomics3/) in the 10 CPTAC cancer types. Using RNA-Seq data, we detected transcripts for all 111 glycosylation enzymes across all 10 CPTAC cancer types. Over half of these enzymes were also identified at the protein level in the corresponding proteomic datasets (Supplementary Fig. S2*A*). Correlation analysis revealed a moderate relationship between RNA and protein expression levels, with median correlation coefficients ranging from 0.31 to 0.49, suggesting additional layers of post-transcriptional regulation (Supplementary Fig. S2*B*). Furthermore, we observed that most of the glycosylation enzymes in the LLO pathway are upregulated in tumor samples compared with NAT samples, with the exception of PDAC and UCEC. In addition, we found that MAN1A1 and MAN2C1 are downregulated in most cancer types, whereas B4GALT1 and B4GALT3–5 are upregulated in most cancer types (Supplementary Fig. S2*C*). The cancer-associated glycoforms shared among the adenocarcinoma group—including BRCA, LUAD, PDAC, UCEC, and CCRCC—are summarized in Supplementary Fig. S2*D* and Supplementary Table S2. Although the number of overlapping glycoforms is limited, we identified six that were consistently and significantly upregulated across BRCA, LUAD, PDAC, and CCRCC but not in UCEC. These six glycoforms originate from three proteins—POSTN, P4HA1, and COL5A2—with half of them derived from the same glycosite, POSTN_N599, carrying either fucosylated-only (only_F) or F + S glycans. POSTN encodes periostin, a secreted ECM protein implicated in tumor progression, extracellular remodeling, and EMT.

The correlation analysis between glycosylation enzymes and glycans in PDAC tumor samples revealed two coherent regulatory patterns, labeled GC.1 (*red*) and GC.2 (*blue*) (Supplementary Fig. S2*E*). GC.1 captures correlations between glycans and Golgi-localized enzymes that mediate late branching and terminal fucosylation/sialylation, whereas GC.2 links glycans to ER/early-stage enzymes involved in precursor assembly, oligosaccharyltransferase transfer, and core N-glycan formation. The glycan type distributions differed markedly: GC.1 was enriched for high-antenna complex glycans (E) with relatively few low-antenna forms, consistent with Golgi-driven terminal elaboration; GC.2 contained all HM glycans (A) and a large fraction (∼37%) of low-antenna glycans (B), reflecting early/ER-biased processing. At the protein level, GC.2 comprised 175 unique proteins, GC.1 comprised 53, and 98 glycoproteins carried glycans from both patterns. Pathway enrichment supported this dichotomy: GC.1-unique proteins were enriched for complement and coagulation cascades and ECM–receptor interaction, indicating a predominance of secreted/extracellular functions. GC.2-associated proteins were enriched for glycan degradation, lysosome, phagosome, and protein processing in ER (with some ECM/complement signal), highlighting internal/ER–lysosomal localization. The overlap set (98 proteins) showed common enrichment for ECM–receptor interaction, complement and coagulation cascades, phagosome, and EMT but was uniquely enriched for focal adhesion, PI3K–AKT signaling, protein digestion and absorption, and proteoglycans in cancer, suggesting roles in cell–cell communication and extracellular signaling.

Collectively, these findings delineate two complementary regulatory networks in PDAC: an ER-centered program dominated by HM/low-antenna glycans, and a Golgi-centered program enriched for high-antenna, F + S complex glycans.

### Glycoproteomics in Molecular Subtypes of PDAC

Molecular subtyping, by integrating multiomics data such as genomics, transcriptomics, and proteomics, enables the further classification of traditionally organ-defined tumors into biologically distinct subgroups. This refined categorization not only reveals the intrinsic heterogeneity within tumors but also lays the foundation for accurate prognosis and personalized therapy, ensuring that targeted therapies and immunotherapies achieve maximum efficacy in the most appropriate patients. As a result, molecular subtyping has become a critical component in advancing precision oncology, accelerating drug development, and optimizing clinical trial design.

**Fig. 3:**
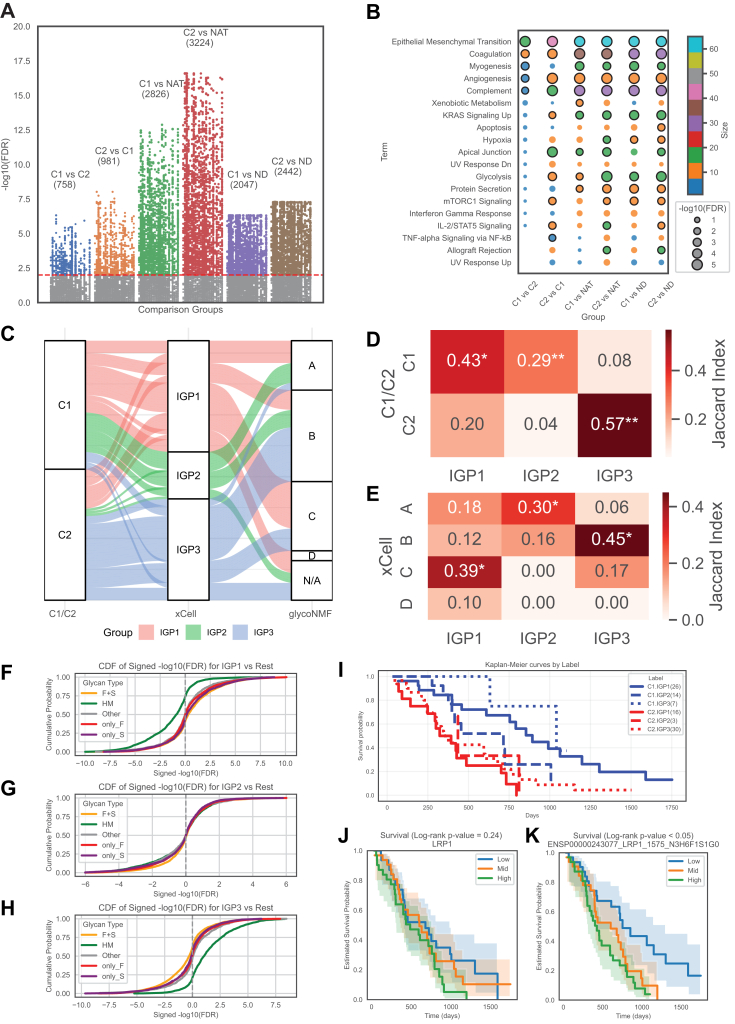
**Glycoproteomics-based clustering of PDAC.***A*, differential expression of glycoforms across molecular (C1/C2) and pathological subtypes (NAT, ND). Each *dot* represents a glycoform, with significance shown as -log10(FDR) on the *y*-axis. Comparison labels indicate the subtraction order (*e.g.*, C1 *versus* C2 = C1-C2, positive values indicate higher abundance in C1). Within each color-coded comparison group, glycoforms are ordered by the chromosomal position of their encoding gene; *vertical stripes* correspond to chromosomes 1 to 22, X and Y. Numbers in *parentheses* denote the count of significantly altered glycoforms (FDR <0.01) in each comparison group. *B*, pathway enrichment of differentially expressed glycoforms across molecular subtypes (C1/C2) and pathological subtypes (NAT and ND). Bubble plot showing enriched pathways identified from glycoforms differentially expressed in each pairwise comparison. “A *versus* B” indicates A − B (positive values = higher in A). Bubble color corresponds to pathway size (number of genes overlapped in the gene set), bubble area to enrichment significance (-log10(FDR)), and the bubble edge color (*black*) indicates significantly enriched pathways with FDR <0.01. *C*, concordance of molecular subtypes identified by C1/C2, xCell, and NMF-based glycoform clustering (IGP clusters). Sankey diagram showing the relationship between molecular subtypes defined by multiomic-based classification (C1/C2, *left*), NMF-based glycoform clustering (IGP1-3, *middle*), and immune/stromal microenvironment classification by xCell (A to D and N/A). Stream widths are proportional to the number of samples shared between categories. Colors represent IGP clusters (IGP1 = *red*, IGP2 = *green*, and IGP3 = *blue*). *D* and *E*, Jaccard similarity between (*D*) C1/C2 subtypes and IGP clusters and (*E*) xCell subtypes and IGP clusters. Each cell shows the Jaccard index (J = ∣A∩B∣/∣A∪B∣) = for the overlap between the two label sets. Significance was assessed using permutation tests (1000 iterations) with Benjamini–Hochberg FDR correction. *Stars* indicate FDR thresholds: ∗∗<0.01 and ∗<0.05. *F*–*H*, cumulative distribution of glycoform differential expression in IGP subtypes *versus* all other samples. Signed -log10(FDR) values are shown for glycoforms in each IGP subtype—(*F*) IGP1, (*G*) IGP2, (*H*) IGP3—compared with the rest. Curves are stratified by glycan type (F + S, HM, only_F, only_S, and Other). Positive values indicate upregulation in the given IGP, and negative values indicate downregulation. *I*, Kaplan–Meier survival curves stratified by combined C1/C2 and IGP subtypes. Survival probability is shown for six groups defined by molecular subtype (C1: *blue*; C2: *red*) and IGP cluster (*solid* = IGP1, *dashed* = IGP2, and *dotted* = IGP3). Numbers in parentheses indicate sample sizes. Differences in survival patterns reflect the combined prognostic impact of molecular and glycoform-based clustering. *J*–*K*, Kaplan–Meier survival curves for LRP1 at the protein and glycosite levels. *J*, survival analysis of LRP1 total protein expression, stratified into low, mid, and high tertiles (log-rank *p* = 0.24, not significant). *K*, survival analysis of the glycoform of ENSP00000243077_LRP1_1575_N3H6F1S1G0, stratified into low, mid, and high tertiles (log-rank *p* < 0.05), showing significant association with patient survival. *Shaded areas* indicate 95% confidence intervals. FDR, false discovery rate; F + S, fucosylated-plus-sialylated; IGP, intact glycopeptide; NAT, normal adjacent tissue; ND, normal duct; NMF, non-negative matrix factorization; PDAC, pancreatic ductal adenocarcinoma.

Multiple molecular subtyping strategies were reported in the original PDAC study (28), including NMF-based proteogenomic clustering, methylation-based subtyping, RNA-based tumor microenvironment (TME) clustering, and three major transcriptomic-based classification schemes (Collisson, Bailey, and Moffitt). In this study, we focused on two representative subtyping results: the NMF-based multiomics clustering (C1/C2), which closely aligns with the Moffitt classical and basal-like transcriptomic subtypes, respectively, and the xCell-based RNA inference clustering, which divides tumors into four groups—A (acinar-high), B (immune cold), C (immune cold), and D (immune hot). These classifications were used to explore how glycoproteomics provides additional insights into PDAC molecular subtypes. Differential expression analysis (Fig. 3*A*) revealed 758 glycoforms upregulated in the C1 subtype compared with C2 and 981 glycoforms upregulated in the C2 subtype compared with C1, highlighting pronounced molecular divergence between the two groups. Comparisons with NATs showed 2826 altered glycoforms in C1 *versus* NAT and 3224 in C2 *versus* NAT, suggesting broad glycosylation changes. Similarly, relative to ND tissues, 2047 and 2442 glycoforms were differentially expressed in C1 and C2 tumors, respectively. Notably, C2 exhibited more extensive alterations, implying a more dysregulated profile.

GSEApy-based ORA revealed hallmark pathway enrichment across differential comparisons based on proteomic profiles (Fig. 3*B*). At the glycoproteome level, glycoforms upregulated in C2 compared with C1 are enriched in EMT, coagulation, angiogenesis, complement, and apical junction together with KRAS signaling up, mTORC1 signaling, glycolysis, and cytokine signaling (IL-2/STAT5, TNF-α/NF-κB), indicating a basal-like/mesenchymal, inflammatory, and metabolically rewired phenotype. In contrast, glycopeptides upregulated in C1 (*versus* C2) are enriched in EMT, coagulation, myogenesis, angiogenesis, and complement, consistent with an ECM/CAF-driven stromal program. The overlap on EMT/coagulation/angiogenesis/complement reflects shared TME axes, but the glycoprotein members and glycosylation sites differ between subtypes. Compared with normal tissues, both C1 and C2 show concordant upregulation of pathways, including EMT, coagulation, myogenesis, angiogenesis, complement, KRAS signaling up, glycolysis, and protein secretion, reflecting the pervasive stromal activation and metabolic reprogramming characteristic of PDAC. Notably, C1 exhibits additional enrichment of xenobiotic metabolism, suggestive of a more epithelial/classical phenotype; in contrast, C2 shows additional enrichment of hypoxia, mTORC1 signaling, and cytokine/allograft-rejection–related pathways, indicating a more pronounced basal-like, inflammatory, and metabolically rewired state. Together with the stronger enrichment of EMT, inflammatory signaling, and metabolic axes in C2 relative to C1, these glycoproteomic-based results support the biological distinctiveness of the NMF-defined subtypes and align C2 with a more aggressive phenotype.

To investigate the intertumor heterogeneity and common pattern of glycoproteomics, we also constructed NMF-based glycoform clustering and found three clusters by the cophenetic score and consensus heatmap (Supplementary Fig. S3, *A* and *B*, Supplementary Table 3). The connections between NMF-based glycoform clustering and the NMF-based multiomics clustering and xCell-based TME clustering are summarized in Figure 3*C*. Heatmaps were employed to present the Jaccard index between glycoform-based intact glycoproteomic subtypes (IGP1–3) and two different tumor subtyping schemes (Fig. 3, *D* and *E*). The overlap analysis demonstrates that the IGPs are biologically meaningful and show concordance with previously established transcriptomic and TME subtypes. Specifically, IGP1 is predominantly associated with the C1 subtype and the immune-cold microenvironment subtype C, suggesting a less aggressive, immune-suppressed profile. IGP2 exhibits a mixed pattern, with partial alignment to xCell subtype A, characterized by acinar-like features. In contrast, IGP3 strongly corresponds to the C2 subtype and xCell subtype B, both of which are associated with a more aggressive tumor phenotype and immune exclusion. These associations highlight the potential of glycoproteomic subtyping to capture clinically and biologically relevant features that complement multiomics tumor classification.

To investigate glycan-specific alterations across NMF-based glycoform subtypes, we performed differential expression analysis of glycoforms in each subtype by comparing tumor samples in that subtype against all other tumor samples. The results were then aggregated into five major glycan types. The cumulative distribution functions of signed -log10(FDR) values for each glycan type are presented in Figure 3, *F*–*H*. These plots reveal a subtype-specific glycosylation pattern: IGP1 is marked by a significant downregulation of HM glycoforms, with approximately 75% being downregulated, whereas IGP3 exhibits the opposite trend, with about 75% of HM glycoforms upregulated. This divergence is consistent with the differential expression patterns of glycosylation enzymes observed across the three NMF-based glycoform clustering groups (IGP1–IGP3), as shown in Supplementary Fig. S3*C*. Notably, glycosylation enzymes were significantly upregulated in the IGP3 group compared with the other clusters, suggesting an overall enhancement of glycan biosynthetic activity. Supporting this, we found that the majority of the altered HM glycoforms in IGP3 were derived from a common set of 166 glycoproteins (Supplementary Fig. S3*D*). Functional enrichment analysis of these glycoproteins revealed significant activation of key oncogenic and cellular reprogramming pathways, including EMT, protein secretion, and KRAS signaling (Supplementary Fig. S3*E*). Together, these findings suggest that the elevated glycosylation machinery in IGP3 compared with IGP1 may contribute to a tumor phenotype characterized by increased secretory activity, mesenchymal transition, and oncogenic signaling, providing mechanistic insights into the functional consequences of glycoform remodeling in cancer. This also implies that although xCell-defined immune clusters B and C are both immune cold, they display distinct glycoform profiles, underscoring the added biological insight provided by glycoform-based subtyping.

While survival differences among the three NMF-based glycoform subtypes (IGP1–3) alone were not statistically significant, integrating these subtypes with NMF-based multiomics clustering (C1/C2) revealed additional prognostic stratification (Fig. 3*I*). Notably, IGP3 within the C2 background was associated with nearly the poorest survival outcomes, whereas C1.IGP3 patients exhibited the most favorable prognosis. These findings suggest that combining proteogenomic and glycoproteomic subtyping enhances the resolution of patient stratification and has the potential to improve prognostic accuracy in PDAC. Considering survival associations may depend on specific glycoforms rather than global glycosylation changes captured by IGP clustering, we re-examined tumor-upregulated glycopeptides, leading to the identification of 42 glycoforms whose alterations were significantly associated with survival (Supplementary Table S3). The vast majority of these were complex glycans containing fucosylation and/or sialylation, whereas only seven (16%) were HM. It is also interesting to find that while all the 14 corresponding proteins are also upregulated in tumors, their protein-level changes alone seldom significantly associated with survival (exceptions: COL12A1 and FBLN5). For example, LRP1 protein expression was not significantly associated with survival (log-rank *p* = 0.24; Fig. 3*J*), whereas one of its glycopeptides, ENSP00000243077_LRP1_1575_N3H6F1S1G0, showed a significant association (log-rank *p* < 0.05; Fig. 3*K*), with the high-expression group exhibiting the poorest prognosis. These findings suggest that certain glycopeptides may serve as better prognostic indicators than their corresponding proteins. However, global glycopeptide alterations encompass not only glycosylation changes related to prognosis but also other information, underscoring the need for improved methods to identify glycosylation subtypes that are optimized for prognosis or survival.

### Glycosylation–Phosphorylation Crosstalk in PDAC

Phosphorylation is well known to play critical roles in the regulation of various signaling pathways (51). However, accumulating evidence suggests that protein glycosylation can also modulate signaling activity (52). To explore the regulatory relationship between glycosylation and phosphorylation, we investigated signaling pathways potentially affected by aberrant glycosylation. Specifically, we analyzed two conditions: intraprotein and interprotein PTM crosstalk between glycosylation and phosphorylation. The former refers to the proteins that have identifications of both phosphorylated and glycosylated peptides. The latter refers to the correlation between all identified glycopeptides and phosphopeptides across different proteins.

**Fig. 4:**
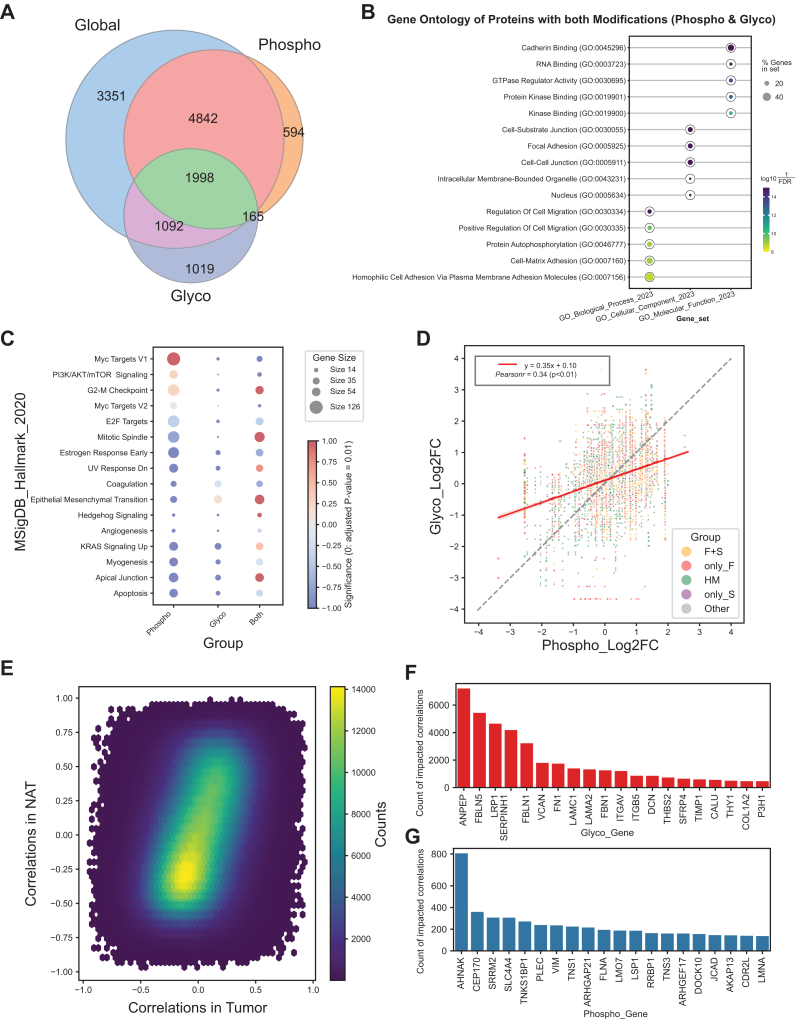
**Glycosylation–phosphorylation crosstalk in PDAC.***A*, overlap among global proteome, phosphoproteome, and glycoproteome datasets. Venn diagram showing the number of proteins identified in the global proteome, phosphoproteome, and glycoproteome datasets. Overlapping regions indicate proteins detected in multiple datasets, including 1998 proteins present in all three (phosphoglycoproteins). Numbers represent protein counts in each category or intersection. *B*, GO enrichment analysis of phosphoglycoproteins (intracrosstalk). Bubble plot showing enriched GO terms for proteins harboring both phosphorylation and glycosylation modifications. Terms are grouped by ontology categories (Biological Process, Cellular Component, and Molecular Function). Bubble size represents the percentage of genes in each term, and color indicates the –log10(FDR) significance level. *C*, comparison of pathway enrichment among phosphoproteins, glycoproteins, and proteins with both phosphorylation and glycosylation modifications. Bubble plot showing significantly enriched MSigDB Hallmark 2020 pathways in each protein group. Bubble size denotes the number of genes in each pathway, whereas bubble color reflects the adjusted *p* value significance level. *Red* indicates significant enrichment, whereas *blue* indicates nonsignificance, with the significance threshold set at an adjusted *p* value of 0.01 (corresponding to 0 on the color bar). *D*, differential expression of phosphosites and glycoforms derived from phosphoglycoproteins. Each point represents a glycopeptide–phosphopeptide pair originating from the same protein name, colored by glycan type. The *red line* denotes the linear regression fit (*y* = 0.35*x* + 0.10), with Pearson's correlation coefficient *r* = 0.34 (*p* < 0.01). *E*, density distribution of intercrosstalk correlations between glycoforms and phosphosites. A Hexbin plot shows the distribution of correlation coefficients between glycopeptides and phosphopeptides originating from different proteins (intercrosstalk). Color intensity indicates the number of pairs within each bin, with warmer colors representing higher pair counts. *F*, top glycoproteins in EMT pathway with the highest number of impacted correlations. *G*, top phosphoproteins in the EMT pathway with the highest number of impacted correlations. EMT, epithelial–mesenchymal transition; FDR, false discovery rate; GO, Gene Ontology; PDAC, pancreatic ductal adenocarcinoma.

Among 4356 glycoproteins and 7630 phosphoproteins analyzed in the PDAC cohort, we found that 2163 proteins harbored both glycosylation and phosphorylation sites (Fig. 4*A*). More than half of these glycoproteins were membrane proteins, based on their CC GO annotations. ORA using GSEApy and GO terms databases (GO_Biological_Process_2023, GO_Cellular_Component_2023, and GO_Molecular_Function_2023) revealed that proteins harboring both phosphorylation and glycosylation are functionally enriched in cell communication, adhesion, and signal transduction pathways. These processes are critical in cancer progression, including cell migration, invasion, and metastasis. The functional dual modification may represent an added layer of regulatory complexity, especially at cell junctions and adhesion sites, making these proteins potential biomarkers or therapeutic targets (Fig. 4*B*). The comparative ORA results of the phosphorylated proteins (Phospho), glycoproteins (Glyco), and dually modified (both glycosylated and phosphorylated, both) proteins on MSigDB_Hallmark_2020 revealed distinct biological roles associated with different classes of post-translationally modified proteins (Fig. 4*C*). Proteins modified by phosphorylation alone were significantly enriched in oncogenic and proliferative pathways, including Myc targets, E2F targets, G2–M checkpoint, and PI3K/AKT/mTOR signaling, highlighting the significant role of phosphorylation in driving tumor cell proliferation and cell cycle progression. In contrast, proteins comodified by both phosphorylation and glycosylation exhibited prominent enrichment in pathways related to tumor progression and microenvironmental remodeling, such as EMT, KRAS signaling, and apical junction formation—hallmarks of invasion, metastasis, and epithelial integrity loss in pancreatic cancer. This dual-modified group also showed enrichment in mitotic spindle and G2–M checkpoint pathways, indicating enhanced proliferative capacity and genomic instability. These observations suggest that dually modified proteins serve as integrative nodes bridging intracellular signaling and extracellular interactions. In contrast, proteins modified by glycosylation alone demonstrated limited enrichment in hallmark pathways, potentially reflecting involvement in broader, less pathway-defined BPs. Collectively, these findings underscore the distinct yet complementary roles of phosphorylation and glycosylation in shaping the molecular landscape of PDAC. To assess the concordance of phosphorylation and glycosylation on the same proteins, we compared the log2FC of glycoforms and phosphosites between tumor and NAT samples (Fig. 4*D*). A moderate yet significant positive correlation was observed (Pearson’s *r* = 0.34, *p* < 0.01), indicating that proteins with increased phosphorylation in tumors also tend to exhibit elevated glycosylation. This correlation was consistent across the five glycan types, with no significant differences observed among them.

To delineate interprotein post-translational crosstalk, we computed correlations between glycoforms and phosphosites originating from different proteins across the PDAC cohort using the paired tumor and NAT samples from 44 patients. Numerous glycoform–phosphosite pairs that were strongly correlated in NAT lost this association in tumor samples, indicating a breakdown of coordinated regulation during tumorigenesis (Fig. 4*E* and Supplementary Table S4). ORA was conducted on gene names derived from glycoforms and phosphosites whose correlations significantly decreased (corr >0.3) in tumor samples. We identified distinct pathway enrichments between glycoproteins and phosphoproteins. Among the glycoproteins, the top-enriched pathways included EMT, coagulation, and complement, reflecting alterations in ECM remodeling and immune modulation. In contrast, the phosphoproteins were predominantly enriched in pathways related to cell cycle and hormone signaling, including mitotic spindle, estrogen response early, and estrogen response late (Supplementary Table S4). These findings suggest that the disruption of coordinated glycosylation and phosphorylation in tumors may contribute to both structural reprogramming of the TME and dysregulation of proliferative and hormonal signaling.

Quantification of the disrupted correlations within the EMT pathway showed that the glycoproteins ANPEP, FBLN5, LRP1, SERPINH1, and FBLN1 account for the largest share of lost associations, consistent with their central roles in ECM remodeling and cell-adhesion networks (Fig. 4*F*). On the paired phosphorylation side, AHNAK, CEP170, SRRM2, SLC44A4, and TNKS1BP1 showed the greatest loss of glycoform partners, pointing to altered control of cytoskeletal organization, RNA processing, membrane transport, and DNA-damage response (Fig. 4*G*). Collectively, these proteins emerge as hubs whose inter-PTM connections are selectively rewired in PDAC, underscoring disrupted glycosylation–phosphorylation coordination as a distinctive molecular feature of this cancer.

We further examined two critical pathways—protein processing in the ER and PI3K–AKT signaling—using the KEGG web tool to visualize the identified glycoproteins and phosphoproteins from the disrupted correlation networks in tumor samples (Supplementary Fig. S4, *A* and *B*). The observed loss of correlation between glycoproteins and phosphoproteins in tumors suggests a functional decoupling between ER glycosylation and phosphorylation-dependent stress signaling. As visualized in the protein processing in the ER pathway, glycoprotein folding and adaptive phosphorylation responses are normally coordinated through tightly regulated mechanisms involving binding immunoglobulin protein, OSTs, and unfolded protein response sensors, such as PERK, ATF6, and IRE1. In tumors, this coordination appears to break down, potentially because of chronic ER stress, dysregulation of glycosylation enzymes, or oncogenic rewiring of phosphorylation networks. This uncoupling may result in impaired protein quality control, accumulation of misfolded glycoproteins, and dysfunctional unfolded protein response signaling—contributing to tumor progression and therapy resistance.

## Discussion

Glycoprotein-Notebook is a novel glycoproteomic database and toolkit designed for IGP analysis in cancer research. The primary focus of the Glycoprotein-Notebook is to aid in the discovery and interpretation of disease-associated glycosites, IGPs, and glycoproteins, thereby complementing existing cancer and glycoproteome data portals. Using datasets from the CPTAC studies and the tools integrated into the Glycoprotein-Notebook platform, we conducted five types of analyses—including differential expression analysis (Fig. 2), glycosylation biosynthesis enzyme analysis (Supplementary Fig. S2, *A*, *C* and S3), glycoform-based subtyping analysis (Fig. 3), survival analysis (Fig. 3, *I* and *J*), and phosphorylation–glycosylation crosstalk analysis (Fig. 4)—to demonstrate its practical utility and highlight the potential of glycopeptides as informative biomarkers for tumor diagnosis, prognosis, and therapeutic targeting.

To investigate the potential role of cancer-associated glycopeptides in the diagnosis of PDAC, we performed differential expression analysis and tumor subtyping. In the differential expression analysis, we revealed that the differential expression of IGPs can provide novel insights into protein alteration in tumor tissues. For example, LAMC1 is a crucial subunit of the laminin gene family, noncollagenous glycoproteins found in basement membranes. Aberrant expression of LAMC1 has been associated with various biological and clinical characteristics in several cancers, including gastric cancer (53), hepatocellular carcinoma (54), renal cell carcinoma (55), colorectal cancer (56), and lung cancer (57). In our cohort, although no significant alterations in LAMC1 were detected at the transcriptomic or proteomic levels, a marked upregulation was observed exclusively in the glycoproteomic data (Fig. 2*F*). This finding underscores the importance of glycoproteomics as a critical complementary layer in multiomics cancer research.

In the tumor subtyping case study, we further demonstrated that glycoform-based clustering was associated with the immune characteristics of the tumor samples (Fig. 3, *C*-E). Integrative clustering of the PDAC proteogenomic cohort resolved two principal tumor-intrinsic states (C1 and C2) and three glycoform-based subclasses (IGP1–3), together highlighting a multilayered architecture of disease heterogeneity. C1 tumors align with the canonical “classical” transcriptomic subtype, retain epithelial features, and are associated with a favorable outcome, whereas C2 mirrors the “basal-like” phenotype, couples hyperproliferative signaling with stromal activation and is linked to poor survival. Superimposing the glycoproteomic strata refined this classification: IGP3 segregated almost exclusively with C2 and further demarcated the worst-prognosis group, whereas IGP1 distinguished an immune-cold branch within C1 that nonetheless retained clinical benefit. Hence, glycoform patterns do not merely echo transcriptomic states but refine them, uncovering clinically—and potentially therapeutically—actionable sublineages.

To investigate the regulatory network of protein glycosylation, we performed PTM crosstalk analysis, in which we provided a comprehensive analysis of protein glycosylation and phosphorylation crosstalk for both intraprotein and interprotein crosstalk analyses. In intraprotein analysis, we found dual modifications preferentially mark pathways that couple proliferative drive with microenvironmental remodeling, including EMT, KRAS signaling, apical-junction integrity, and mitotic-spindle control. These data position the dual-modified cohort as integrative hubs that translate intracellular kinase activity into ECM interactions and *vice versa*, thereby potentiating both tumor growth and metastatic competence. Our interprotein analysis uncovered a pronounced breakdown of glycophospho correlation in tumors relative to NAT. Glycoproteins such as ANPEP, FBLN5, LRP1, SERPINH1, and FBLN1—key constituents of the ECM and adhesion machinery—lose the majority of their phosphorylation partners. Reciprocally, phosphoproteins, such as AHNAK, CEP170, SRRM2, SLC44A4, and TNKS1BP1, forfeit glycoform linkages that typically coordinate cytoskeletal regulation, RNA splicing, membrane transport, and DNA-damage signaling to the extracellular milieu. The selective rewiring of these hubs indicates that tumor cells decouple surface glycan remodeling from intracellular kinase networks, a strategy that may facilitate immune evasion, matrix reorganization, and unchecked proliferation. Taken together, our findings delineate a PDAC-specific “PTM dyscoordination” signature in which phosphorylation drives classical oncogenic programs, whereas glycosylation—particularly in combination with phosphorylation—modulates the tumor–stroma interface. Disrupted crosstalk between these PTMs highlights actionable vulnerabilities: restoring PTM coordination or selectively targeting dual-modified hubs could provide novel therapeutic avenues. Future work integrating site-specific PTM stoichiometry with functional perturbations will be essential to unravel the causal hierarchy within this regulatory web and to translate these molecular insights into clinically tractable strategies for PDAC.

In summary, we developed a comprehensive data analysis pipeline for IGP analysis in cancer studies. The modules designed in the Glycoprotein-Notebook make it easy for users to reproduce the analysis and understand the results. Although the current version of Glycoprotein-Notebook includes only CPTAC data, it is readily extensible to support other cohort-based glycoproteomics studies.

## Declaration of Generative Artificial Intelligence and Artificial Intelligence–Assisted Technologies in the Writing Process

During the preparation of this work, the author(s) used ChatGPT in order to assist with language clarity and grammar checks. After using this tool/service, the author(s) reviewed and edited the content as needed and take(s) full responsibility for the content of the published article.

## Data Availability

Glycoprotein-Notebook is readily accessible *via* https://glycoprotein-notebook.org. Users can explore all functions of Glycoprotein-Notebook by interactively running the Jupyter notebooks locally, following the tutorial for guidance. The raw proteomic data files generated during this study are available at the Proteomic Data Commons (https://pdc.cancer.gov/pdc/). Genomic, epigenomic, and transcriptomic data generated for this publication are available at the Genomic Data Commons (https://gdc.cancer.gov/). All processed data tables and precomputed results are stored at Zenodo ( https://zenodo.org/records/14019975).

## Supplemental data

This article contains supplemental data.

## Conflict of Interest

H. Z. is a founder and CSO of CompleteOmics in a relationship overseen by Johns Hopkins University. The other authors declare no competing interests.
