# Supplementary material for: Glycoprotein-Notebook: A Pan-Cancer Glycoproteomic Database and Toolkit for Analysis of Protein Glycosylation Changes Associated With Cancer Phenotypes
Source: Mol Cell Proteomics. 2025 Oct 13;24(11):101089. doi: 10.1016/j.mcpro.2025.101089 (PMC12681939; doi:10.1016/j.mcpro.2025.101089)

Figure S1. Comparison between Phospho- and Glyco-enriched methods in PDAC samples

A. Glycan Type Distribution in Glyco-Enriched Data (G-Enrich)

B. Glycan Type Distribution in Phospho-Enriched Data (P-Enrich)

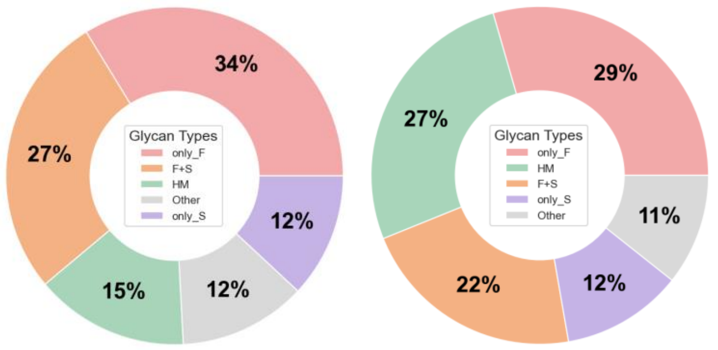

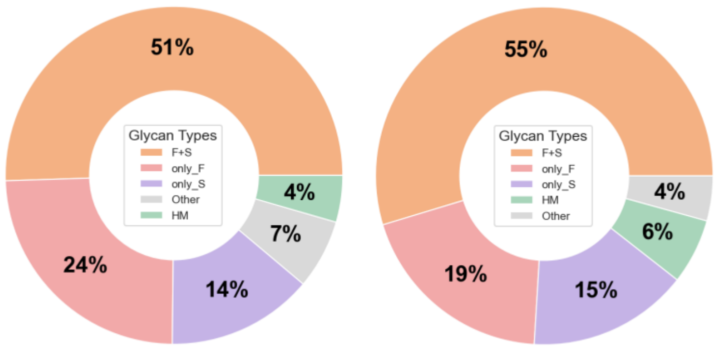

ID in > 1 sample ID in ≥ 50% samples ID in > 1 sample ID in ≥ 50% samples

C.

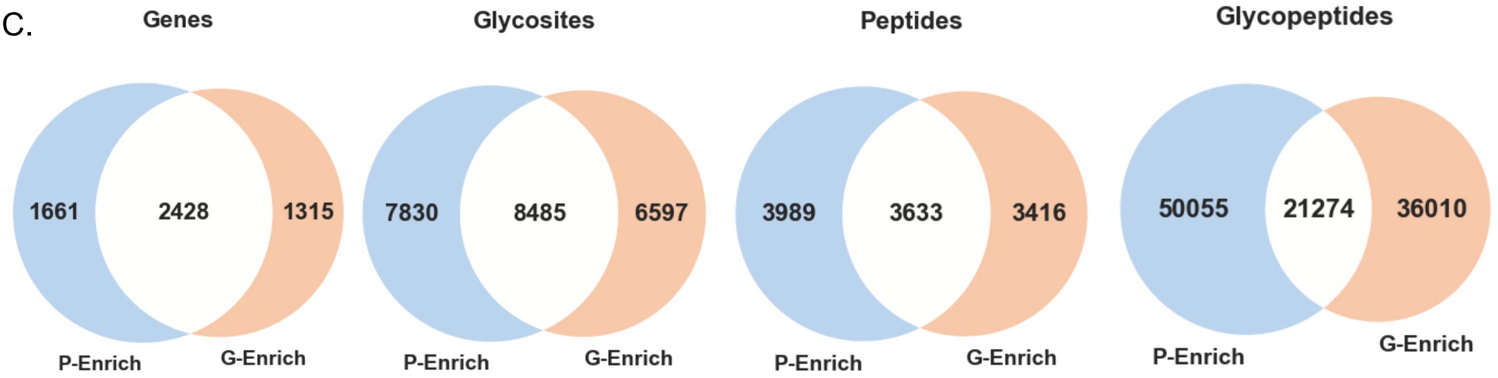

D.

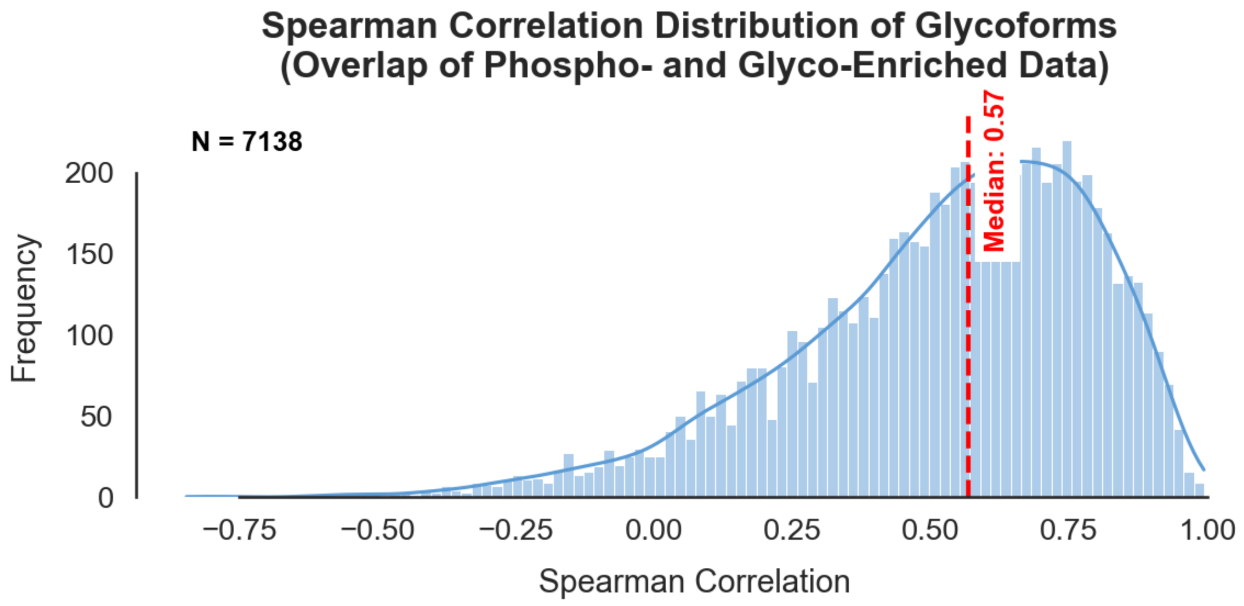

Figure S2. Glycosylation enzymes and shared cancer associated glycoforms crossing cancers

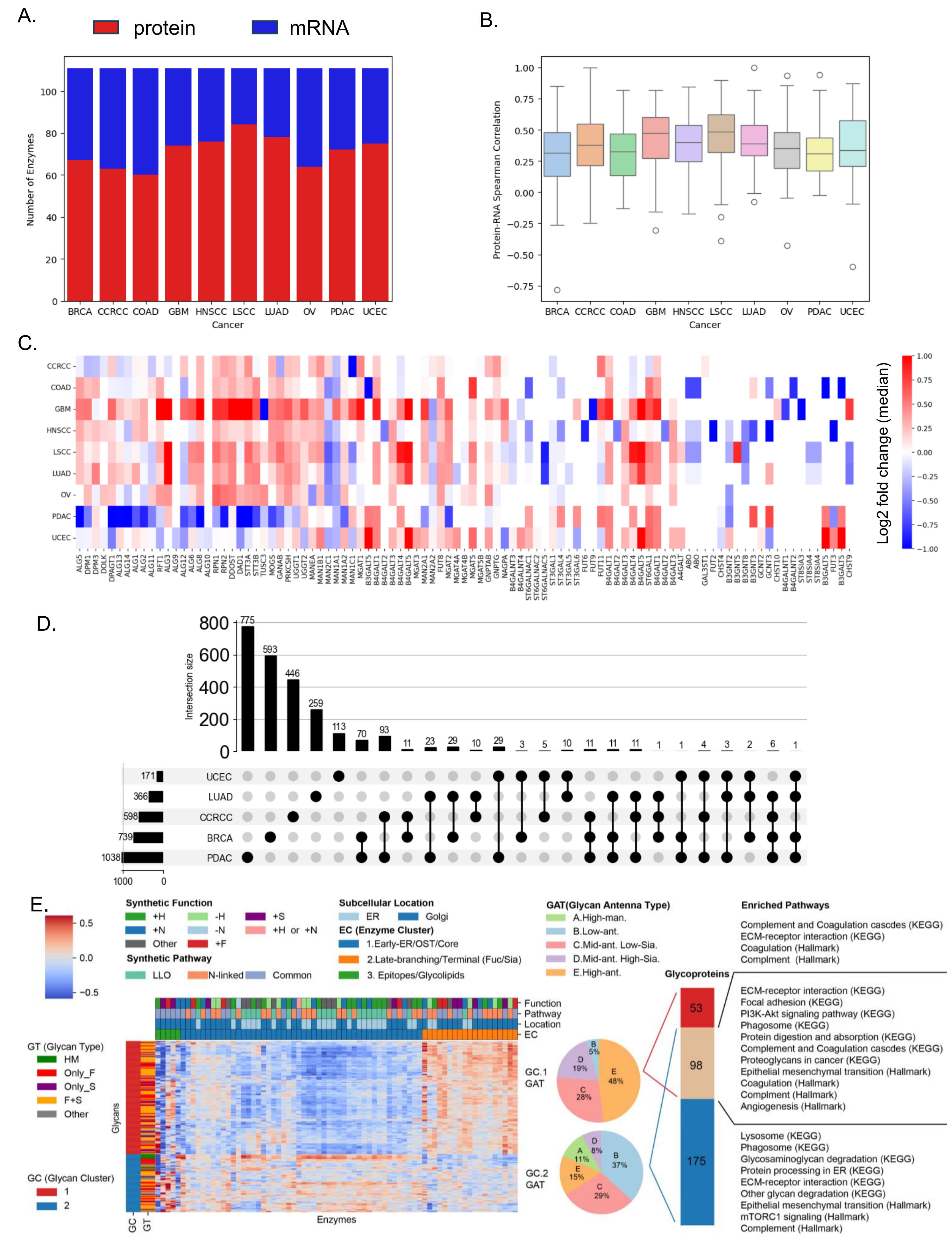

Figure S3. Glycoproteomics analysis in molecular subtyping

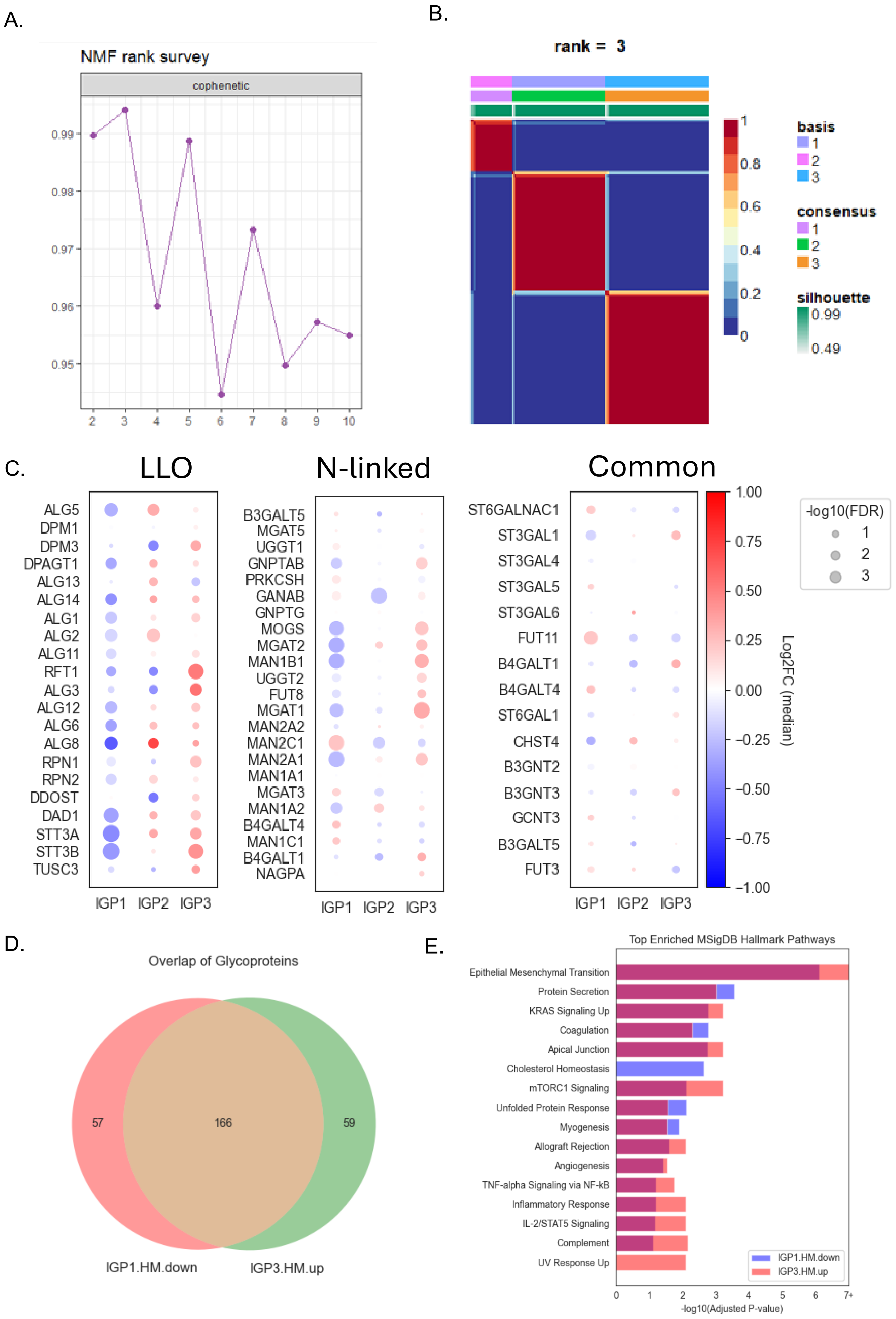

Figure S4 KEGG Pathways visualization

A.

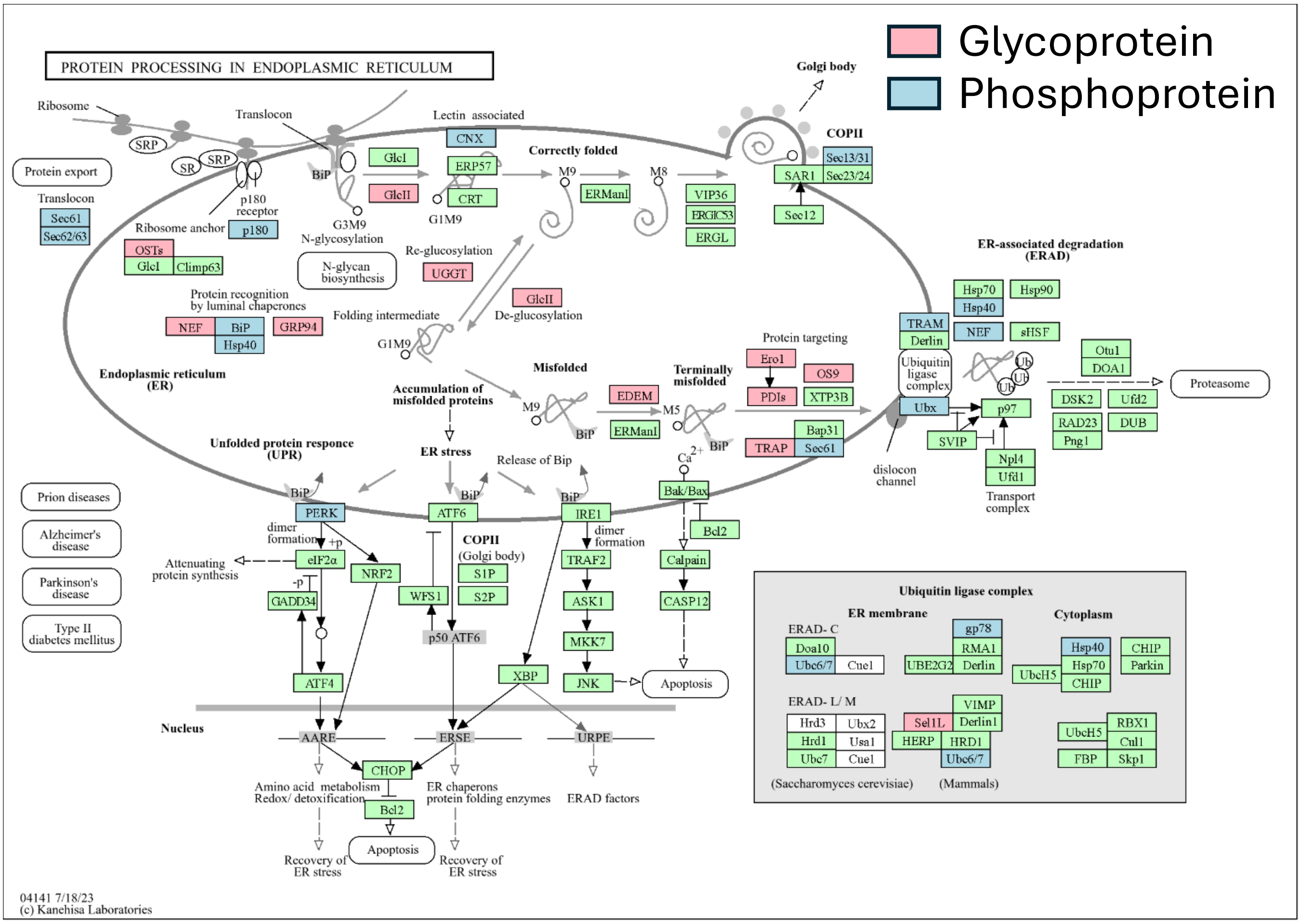

B.

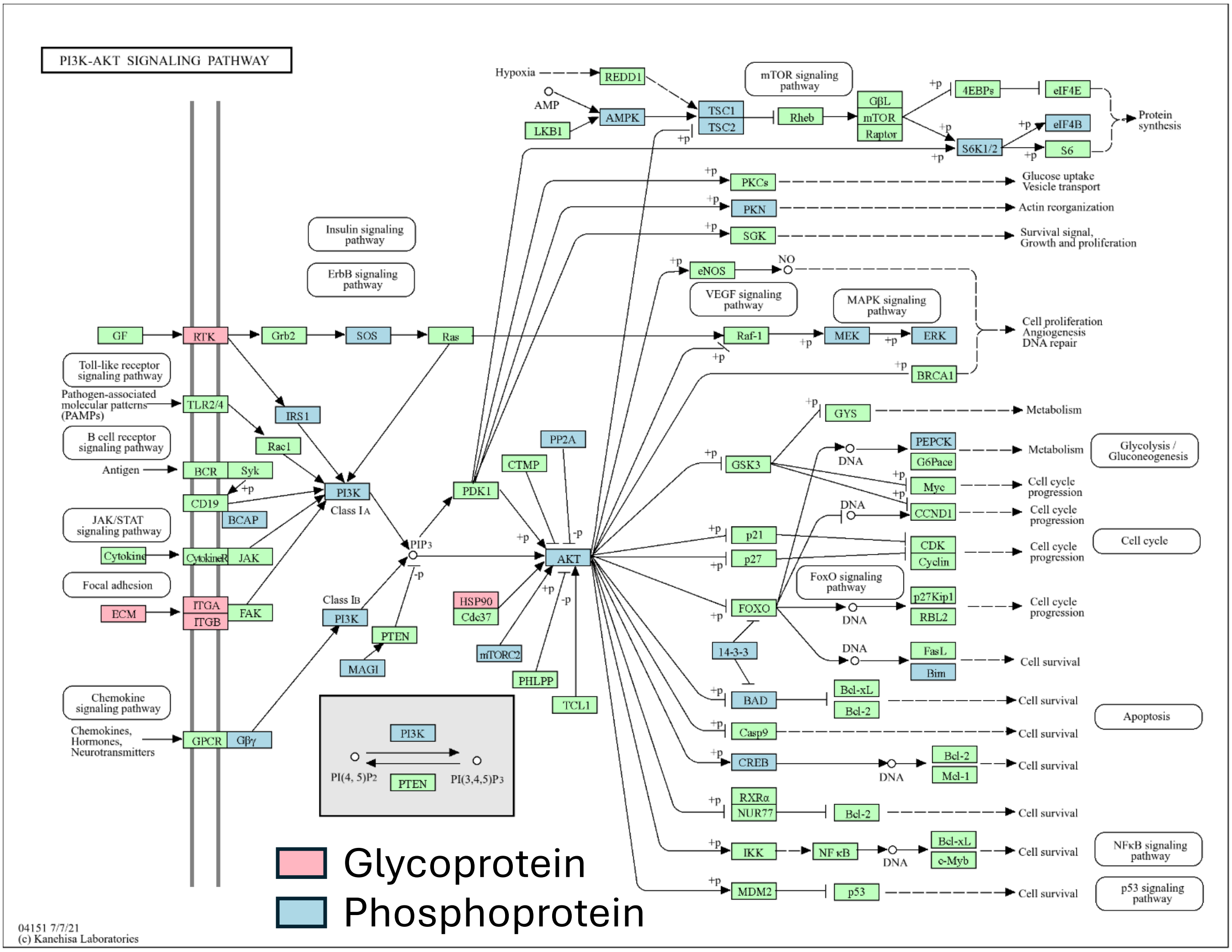

Supplement: Supplementary Figures [file mmc1.pdf]
