# Supplementary material for: Glycoprotein-Notebook: A Pan-Cancer Glycoproteomic Database and Toolkit for Analysis of Protein Glycosylation Changes Associated With Cancer Phenotypes
Source: Mol Cell Proteomics. 2025 Oct 13;24(11):101089. doi: 10.1016/j.mcpro.2025.101089 (PMC12681939; doi:10.1016/j.mcpro.2025.101089)
Supplement: Supplementary File 1 [file mmc2.pptx]

## Slide 1
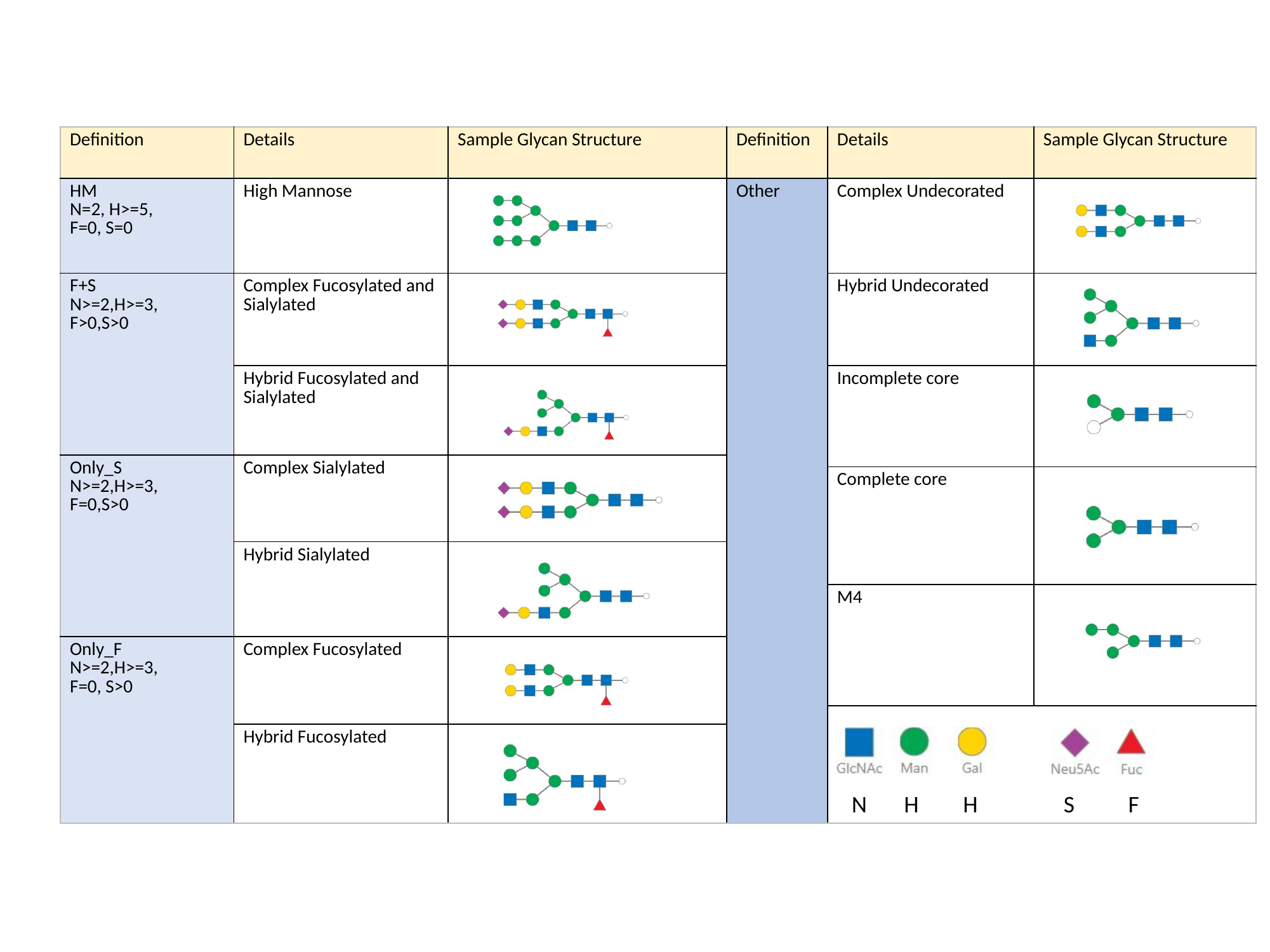

| Definition | Details | Sample Glycan Structure | Definition | Details | Sample Glycan Structure |
| --- | --- | --- | --- | --- | --- |
| HM N=2, H>=5, F=0, S=0 | High Mannose | | Other | Complex Undecorated | |
| F+S N>=2,H>=3, F>0,S>0 | Complex Fucosylated and Sialylated | | | Hybrid Undecorated | |
| | Hybrid Fucosylated and Sialylated | | | Incomplete core | |
| Only\_S N>=2,H>=3, F=0,S>0 | Complex Sialylated | | | | |
| S N>=2,H>=3, F=0,S>0 | Complex Sialylated N>=4,H>=5, F=0,S>0 | | | Complete core | |
| | Hybrid Sialylated | | | | |
| | | | | M4 | |
| Only\_F N>=2,H>=3, F=0, S>0 | Complex Fucosylated | | | | |
| F N>=2,H>=3, F=0, S>0 | Complex Fucosylated N>=4,H>=5, F>0,S=0 | | | | |
| | Hybrid Fucosylated | | | | |
N
H
H
S
F

## Slide 2
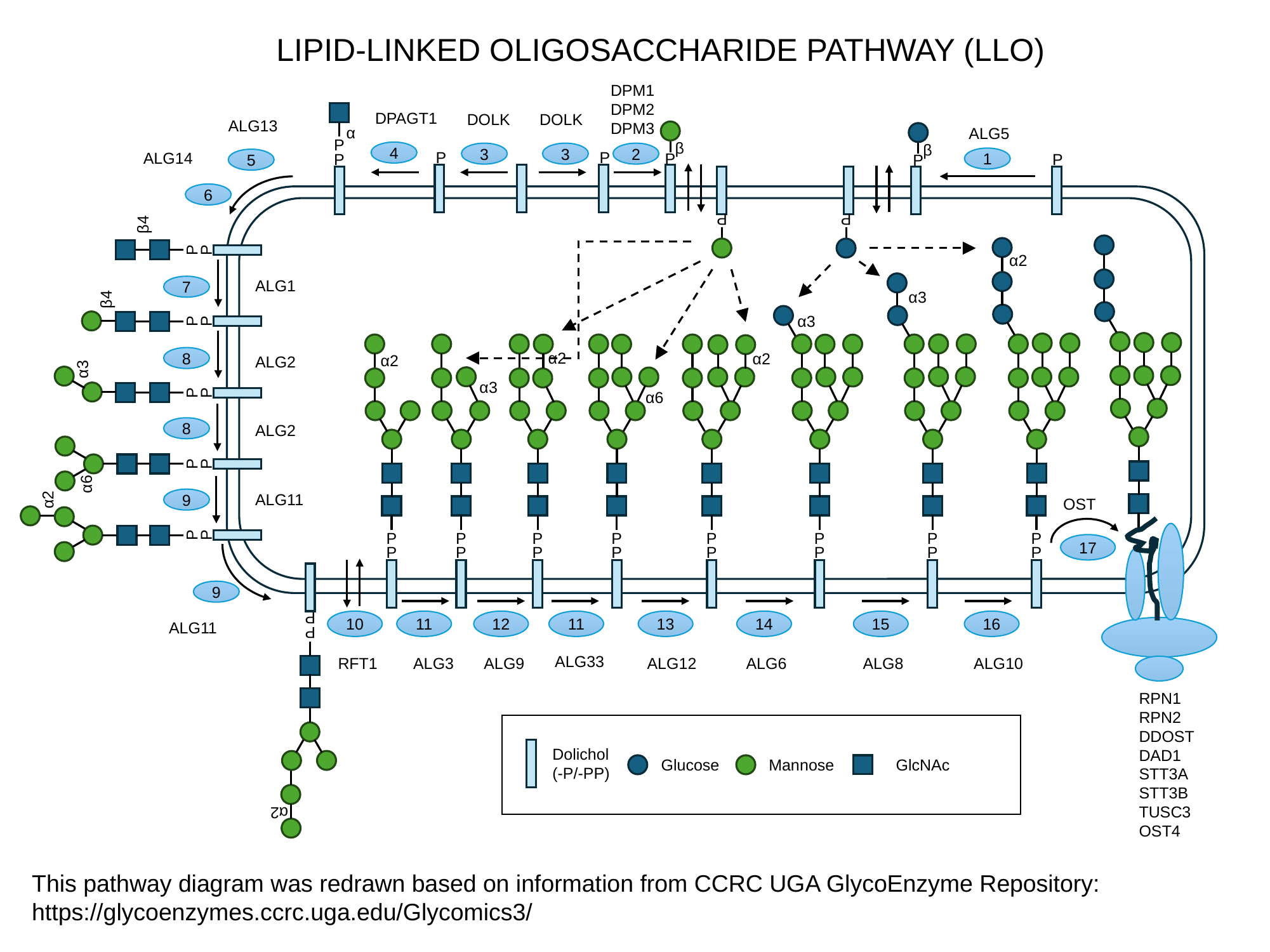

LIPID-LINKED OLIGOSACCHARIDE PATHWAY (LLO)
DPM1
DPM2
DPM3
P
P
DPAGT1
DOLK
DOLK
ALG13
α
ALG5
β
β
4
P
P
3
3
2
ALG14
P
P
P
1
5
6
P
P
β4
P
P
α2
ALG1
7
α3
β4
α3
P
P
P
P
α2
α2
α2
8
ALG2
α3
α3
P
P
α6
ALG2
8
P
P
α6
ALG11
α2
OST
9
P
P
P
P
P
P
P
P
P
17
P
P
P
P
P
P
P
9
10
11
12
11
13
14
15
16
ALG11
ALG33
RFT1
ALG3
ALG9
ALG12
ALG6
ALG8
ALG10
P
P
RPN1
RPN2
DDOST
DAD1
STT3A
STT3B
TUSC3
OST4
Dolichol
(-P/-PP)
Glucose
Mannose
GlcNAc
α2
This pathway diagram was redrawn based on information from CCRC UGA GlycoEnzyme Repository: https://glycoenzymes.ccrc.uga.edu/Glycomics3/

## Slide 3
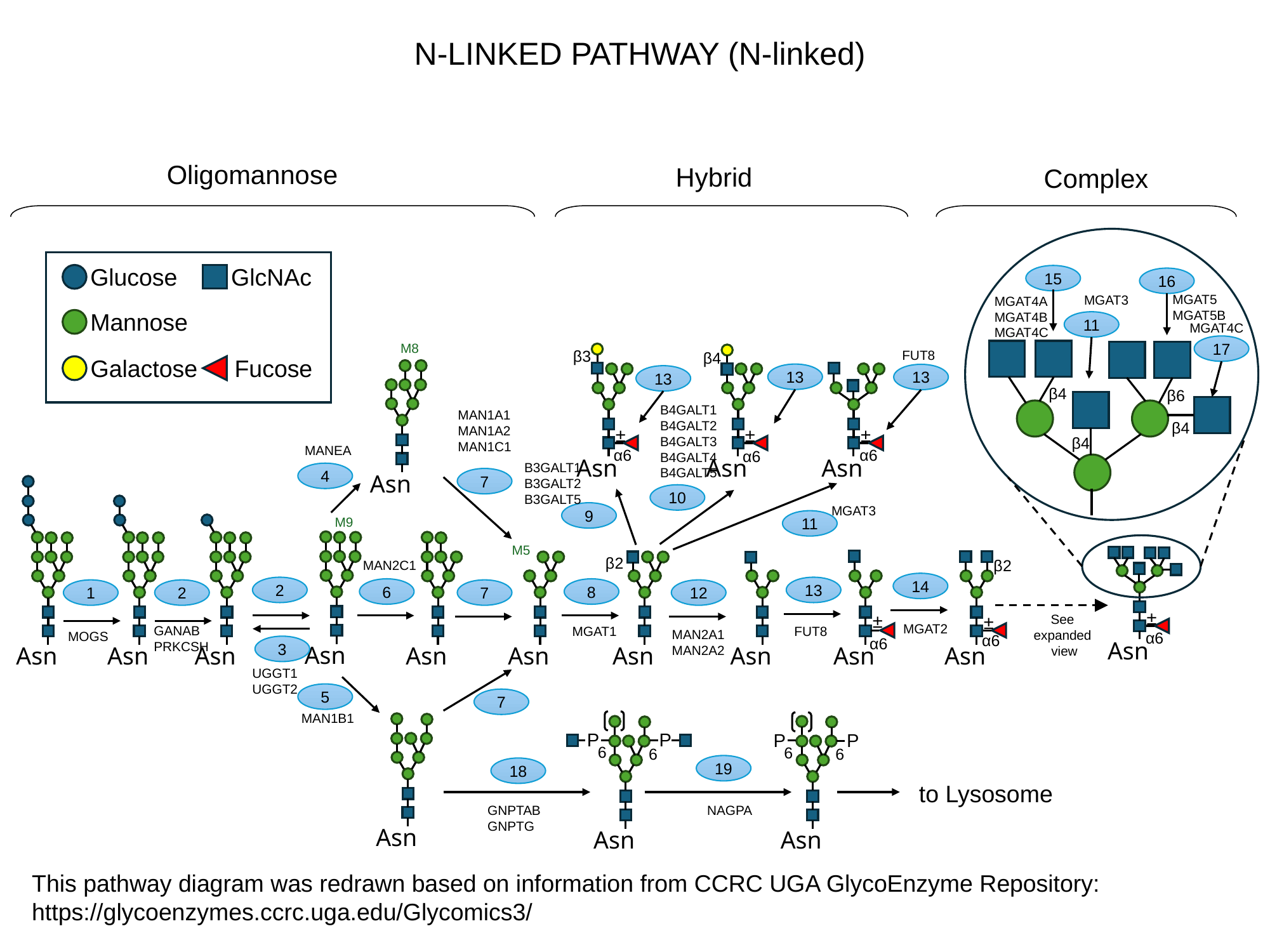

N-LINKED PATHWAY (N-linked)
Oligomannose
Hybrid
Complex
15
16
MGAT5
MGAT5B
MGAT3
MGAT4A
MGAT4B
MGAT4C
11
MGAT4C
17
β4
β6
β4
β4
Glucose
GlcNAc
Mannose
M8
β3
FUT8
β4
Galactose
Fucose
Asn
13
13
13
B4GALT1
B4GALT2
B4GALT3
B4GALT4
B4GALT5
MAN1A1
MAN1A2
MAN1C1
±
±
±
MANEA
α6
α6
α6
Asn
Asn
Asn
B3GALT1
B3GALT2
B3GALT5
4
7
Asn
10
MGAT3
9
M9
11
M5
β2
β2
MAN2C1
14
2
13
6
8
1
2
7
12
±
±
±
See
expanded
view
MGAT2
GANAB
PRKCSH
MGAT1
FUT8
MAN2A1
MAN2A2
MOGS
α6
α6
α6
Asn
Asn
3
Asn
Asn
Asn
Asn
Asn
Asn
Asn
Asn
UGGT1
UGGT2
5
7
MAN1B1
Asn
P
P
P
P
6
6
6
6
19
18
to Lysosome
NAGPA
GNPTAB
GNPTG
Asn
Asn
This pathway diagram was redrawn based on information from CCRC UGA GlycoEnzyme Repository: https://glycoenzymes.ccrc.uga.edu/Glycomics3/

## Slide 4
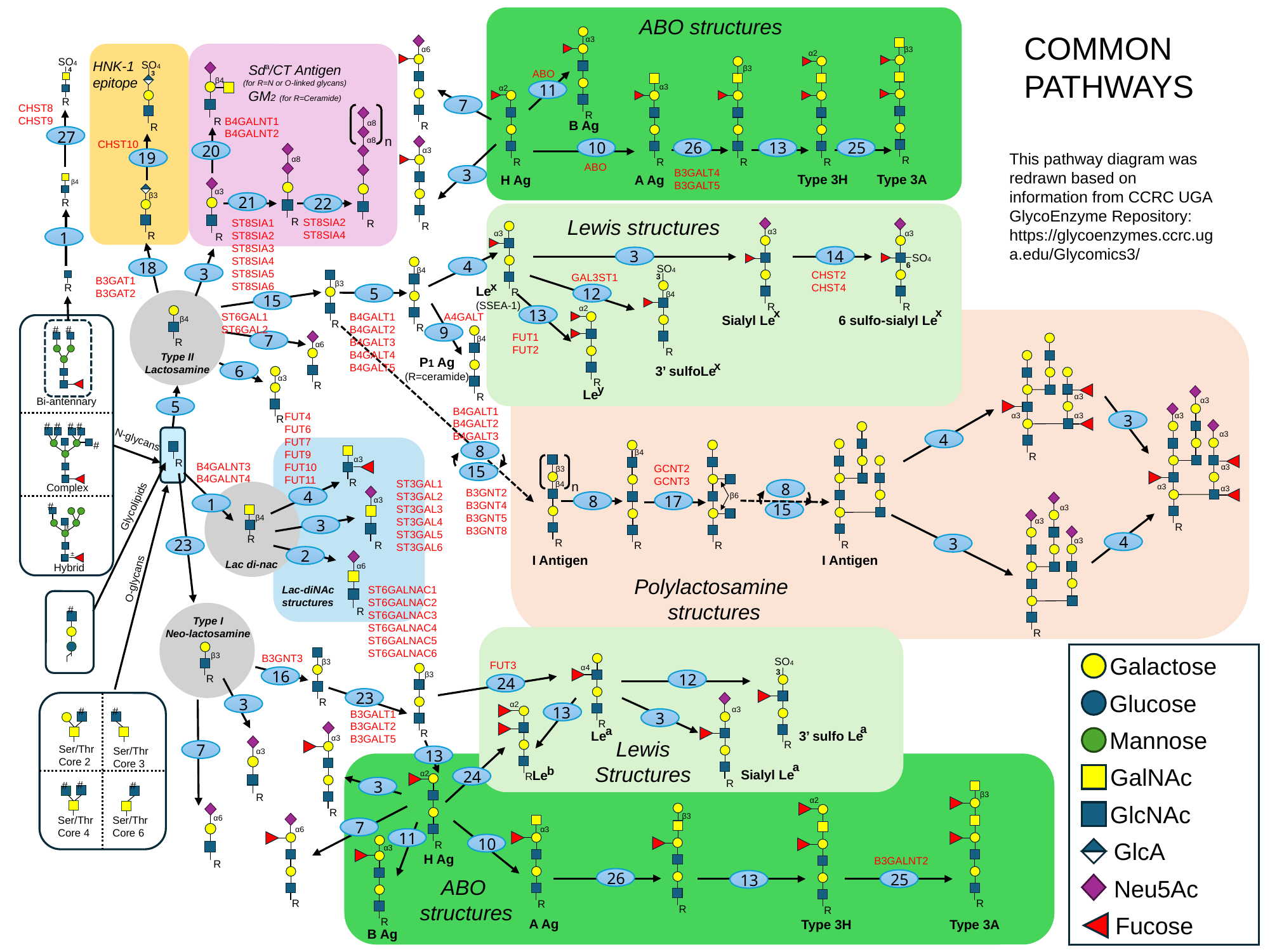

ABO structures
COMMON
PATHWAYS
R
α3
α6
R
β3
α2
SO4
4
R
HNK-1
epitope
SO4
3
R
Sd /CT Antigen
(for R=N or O-linked glycans)
GM2 (for R=Ceramide)
a
β3
β4
R
ABO
11
α3
α2
R
R
R
R
R
7
CHST8
CHST9
α8
n
α8
R
B4GALNT1
B4GALNT2
B Ag
27
CHST10
10
26
13
25
α3
R
20
α8
R
19
This pathway diagram was redrawn based on information from CCRC UGA GlycoEnzyme Repository: https://glycoenzymes.ccrc.uga.edu/Glycomics3/
ABO
3
B3GALT4
B3GALT5
Type 3H
Type 3A
H Ag
A Ag
β4
R
α3
R
β3
R
21
22
Lewis structures
ST8SIA2
ST8SIA4
ST8SIA1
ST8SIA2
ST8SIA3
ST8SIA4
ST8SIA5
ST8SIA6
R
6
R
SO4
α3
R
α3
α3
1
14
3
4
18
β4
R
SO4
3
CHST2
CHST4
GAL3ST1
3
B3GAT1
B3GAT2
β3
R
R
x
Le
(SSEA-1)
5
12
β4
R
15
α2
R
x
6 sulfo-sialyl Le
13
x
Sialyl Le
ST6GAL1
ST6GAL2
β4
R
A4GALT
B4GALT1
B4GALT2
B4GALT3
B4GALT4
B4GALT5
#
#
9
β4
R
FUT1
FUT2
7
α6
R
R
Type II
Lactosamine
P1 Ag
(R=ceramide)
x
3’ sulfoLe
6
α3
R
y
Le
R
α3
Bi-antennary
α3
5
B4GALT1
B4GALT2
B4GALT3
FUT4
FUT6
FUT7
FUT9
FUT10
FUT11
3
α3
α3
α3
#
#
#
#
α3
4
N-glycans
#
8
15
R
β4
α3
R
B4GALNT3
B4GALNT4
GCNT2
GCNT3
α3
β3
ST3GAL1
ST3GAL2
ST3GAL3
ST3GAL4
ST3GAL5
ST3GAL6
n
β4
8
15
Complex
α3
α3
B3GNT2
B3GNT4
B3GNT5
B3GNT8
4
β6
α3
R
8
17
1
R
#
Glycolipids
α3
R
β4
3
α3
±
4
3
23
α3
R
R
R
R
2
±
I Antigen
I Antigen
α6
R
Lac di-nac
Hybrid
O-glycans
Polylactosamine
structures
Lac-diNAc
structures
ST6GALNAC1
ST6GALNAC2
ST6GALNAC3
ST6GALNAC4
ST6GALNAC5
ST6GALNAC6
#
Type I
Neo-lactosamine
β3
R
Galactose
B3GNT3
SO4
3
R
β3
α4
R
FUT3
16
β3
12
24
Glucose
23
3
R
α2
R
13
#
#
α3
R
B3GALT1
B3GALT2
B3GALT5
3
a
3’ sulfo Le
a
Le
Mannose
R
α3
R
Lewis
Structures
7
α3
R
Ser/Thr
Core 2
Ser/Thr
Core 3
13
a
Sialyl Le
GalNAc
b
Le
24
α2
3
#
#
#
R
β3
α2
R
GlcNAc
α6
R
β3
R
Ser/Thr
Core 6
Ser/Thr
Core 4
7
α3
α6
R
11
10
GlcA
R
α3
R
H Ag
B3GALNT2
26
25
13
Neu5Ac
ABO
structures
R
Fucose
A Ag
Type 3H
Type 3A
B Ag
